# Design, molecular docking, and molecular dynamics of thiourea-iron (III) metal complexes as NUDT5 inhibitors for breast cancer treatment

**DOI:** 10.1016/j.heliyon.2022.e10694

**Published:** 2022-09-19

**Authors:** Ruswanto Ruswanto, Tita Nofianti, Richa Mardianingrum, Dini Kesuma, Siswandono

**Affiliations:** a Faculty of Pharmacy, Universitas Bakti Tunas Husada, Tasikmalaya, Indonesia; b Department of Pharmacy, Universitas Perjuangan, Tasikmalaya, Indonesia; c Faculty of Pharmacy, Universitas Surabaya, Surabaya, Indonesia; d Faculty of Pharmacy, Universitas Airlangga, Surabaya, Indonesia

**Keywords:** Cancer, Iron (III), Metal complex, NUDT5, Thiourea

## Abstract

In research, anticancer agents, such as thiourea derivative compounds, and metal complexes, such as those complexed with iron (III) metal, are often studied. The metal complexes are presumably more active than thiourea derivatives as free ligands; some negative effects may be reduced. The computational studies used in this study involved molecular docking with AutoDock and molecular dynamics (MD) simulations using Desmond to evaluate the stability of the interactions. The docking and MD analysis results showed that compounds **2** and **6** had stable interactions with NUDIX hydrolase type 5 (NUDT5)—one of the therapeutic targets for breast cancer—where they had the lowest root mean square deviation (RMSD) and root mean square fluctuation (RMSF) values compared to the other compounds. Together, these compounds are anti-breast cancer drug candidates.

## 1 Introduction

By 2020, approximately 2.3 million women were diagnosed with breast cancer, with 685,000 deaths globally. By the end of 2020, about 7.8 million women have been diagnosed with breast cancer in the past five years, making it one of the most common cancers worldwide. Breast cancer occurs in women residing in any country, at any age after puberty, but the occurrence rates increase later in life [21, 30, 41]. Moreover, the available chemotherapeutic agents have many adverse side effects; therefore, it is necessary to develop a safe anti-breast cancer drug [27].

**Figure 1:**
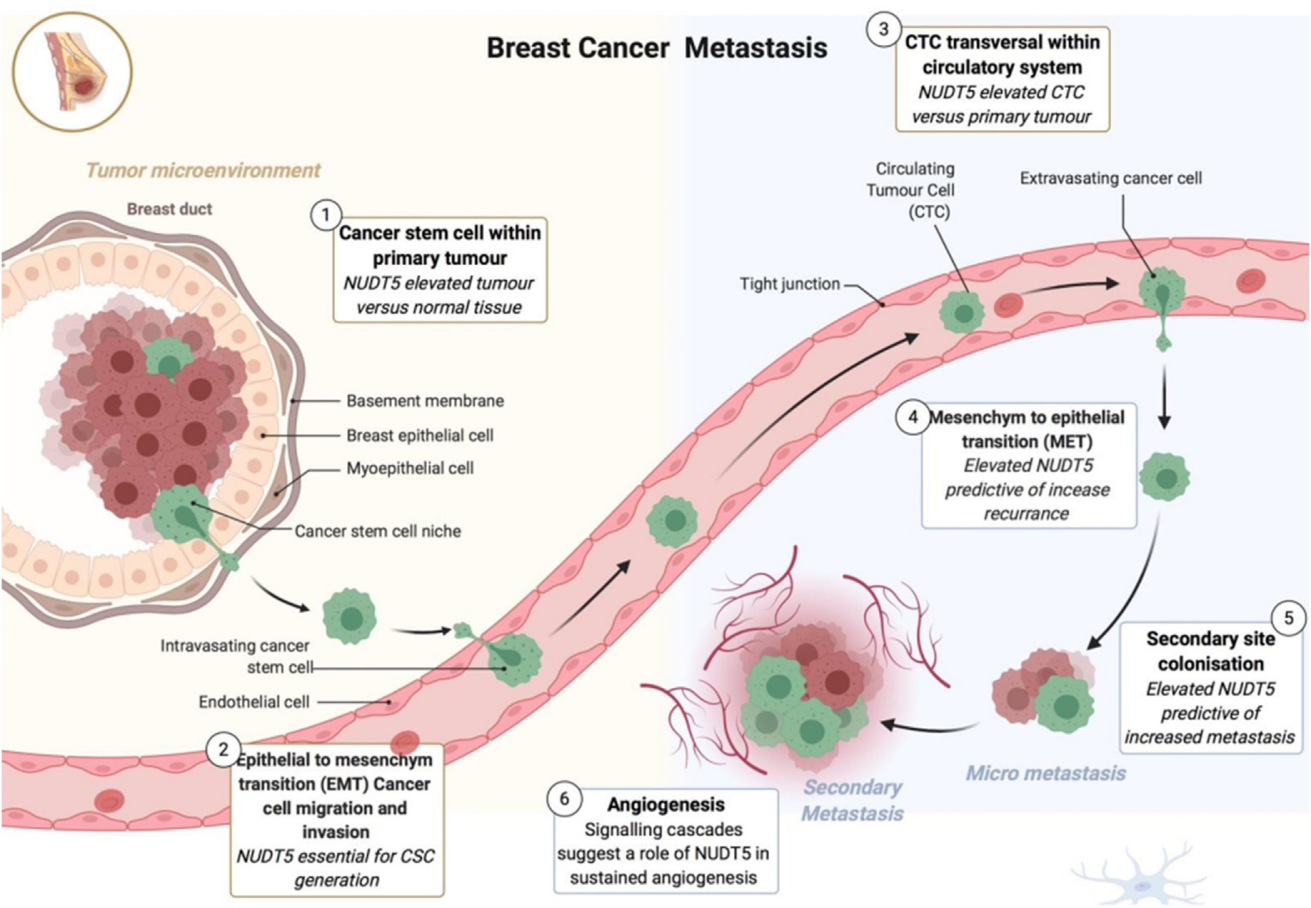
The role of NUDT5 in breast cancer metastasis. Model showing the multiple roles and indications for a key role of NUDT5 in aggressive breast cancer. (1) NUDT5 is elevated in tumor versus normal breast cancer tissue. (2) NUDT5 is essential for breast cancer stem cell (BCSC) generation and maintenance. (3) NUDT5 is highly expressed in circulating tumor cells (CTCs). (4 and 5) Elevated levels of NUDT5 are associated with increased levels of recurrence and metastasis in patients suggesting a role in mesenchyme to epithelial transition and secondary site colonization. And finally, (6) analysis of the gene expression changes occurring in BCSC in 3D cell culture suggests a role of NUDT5 in angiogenesis.

NUDIX hydrolase type 5 (NUDT5, also called NUDIX5) is a significant target for the development of breast cancer drugs. NUDT5 is a type of ADP-ribose pyrophosphatase and a cellular nucleotide metabolizing enzyme. The expression of NUDT5 has been linked to chromosomal remodeling, cell adhesion, the maintenance of cancer stem cells, and the epithelial-to-mesenchymal transition in breast cancer cells, according to earlier research [46]. One important family of nucleotide metabolic enzymes is the NUDIX hydrolase group. Recently, NUDT5 was discovered to function as a rheostat for hormone-dependent gene regulation and proliferation in breast cancer cells. NUDT5 has been linked to the metabolism of ADP-ribose and 8-oxo-guanine [26]. The role of NUDT5 in breast cancer metastasis [42] can be seen in Figure 1.

In drug discovery and development, thiourea-derived compounds have been reported to have anti-tumour [14, 18, 28, 43, 44], antibacterial, antimicrobial, and anti-tuberculosis activity [6, 7, 16, 36, 39, 40], as well as being soluble epoxide hydrolase inhibitors and antiviral [8]. Recently, metal complexes of different transition metals (e.g., platinum, iron, ruthenium, and cobalt) have become the preferred candidates for developing treatments for other cancer types [1, 11, 15, 22, 23].

Triapine was examined in phase I trials in another investigation on thiourea derivatives. Some thiosemicarbazones, like triapine, have biological action because they may form tridentate chelates with transition metal ions attached to oxygen, nitrogen, and sulfur (O–N–S) atoms or sulphur and two nitrogen atoms (S–N–N). Thiosemicarbazones with pyridine rings create S–N–N tridentate systems, and the metal complexes of these systems have attracted a lot of attention due to their biological activity. Lipophilicity, which regulates the rate of cell entry, is impacted by coordination. In addition, some adverse effects may be lessened, and metal complexes may be more active than thiourea derivatives as free ligands [2, 47]. Iron complexes, by contrast, exhibit much stronger anticancer activities than free ligands do. The reactivity of the complexes may explain the cytotoxicity and anticancer activity of thiosemicarbazone-iron (III) complexes. Many processes, including intercalation, DNA and RNA inhibition, and other biomolecular interactions, are studied to elucidate the performance of these drugs [35]. Given their physical and biological properties, iron–salen complexes have been studied since 1931, and it has been established that salen complexes of Fe (III) exert anticancer effects on MCF-7 cells. Previously, we synthesized several derivatives of thiourea compounds and studied their activity in several cancer cell lines, including MCF-7, T47D, WiDr, and HeLa [31, 32, 34].

**Figure 2:**
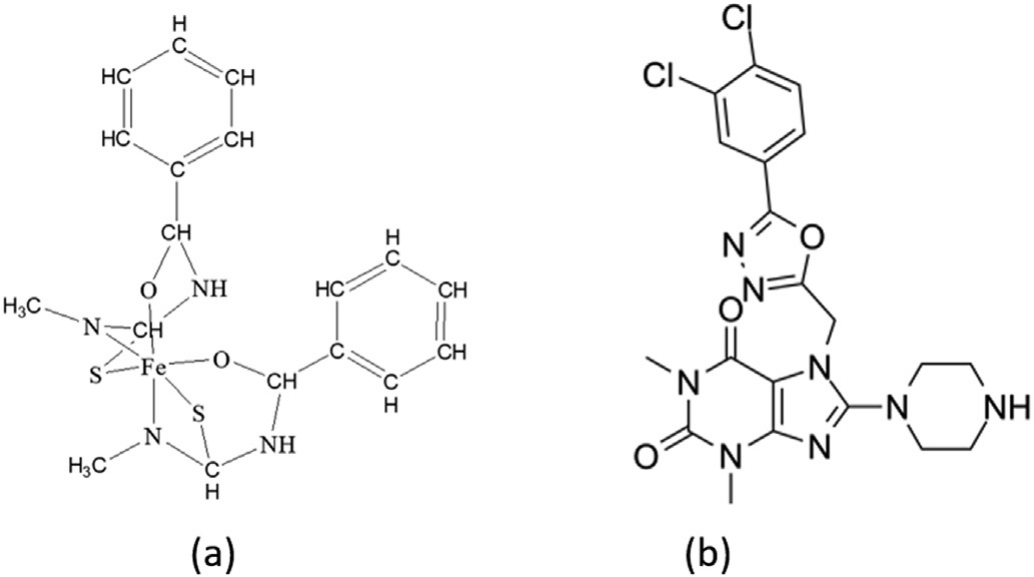
The thiourea-iron (III) metal complex (a) and TH5427 as NUDT5 inhibitor (b).

**Figure 3:**
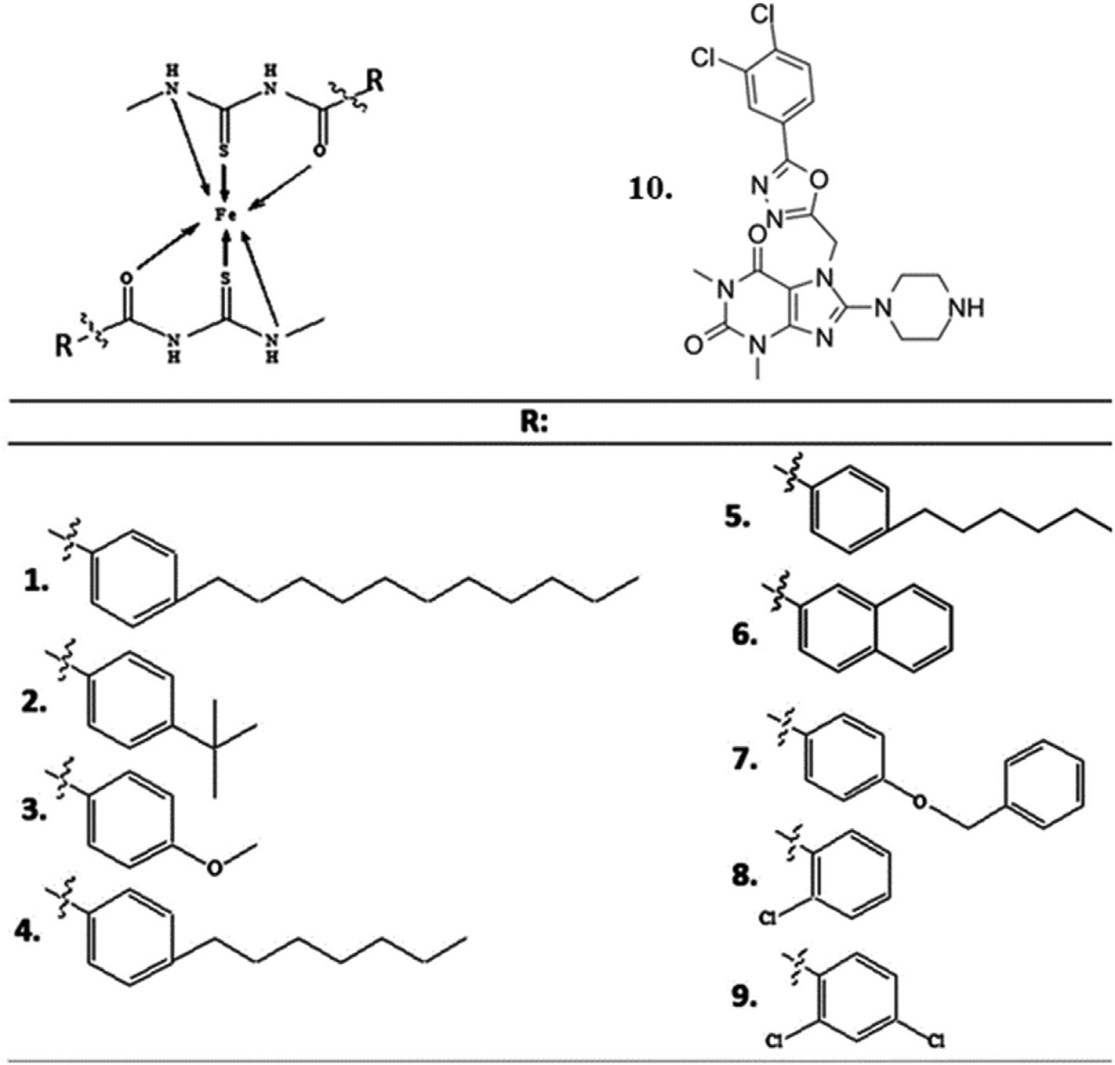
Structure of iron (III) metal complex compounds.

In the present study, to increase anticancer activity, from Figures 2a and 2b, we can see that thiourea-iron (III) metal complexes are almost similar to TH5427, which has not been clinically proven to be active as a NUDT5 inhibitor, so in the research that we designed compounds as iron (III)-thiourea complexes, as shown in Figure 3.

Here, we designed and conducted computational studies of several complexes of iron metal (III) and thiourea-derived compounds with NUDT5 to assess the possibility of developing thiourea compounds as therapeutic drug candidates for breast cancer through NUDT5 inhibition. The metal complex design's choice of substituent was made because it will impact the lipophilic, electronic, and steric properties, which are projected to result in changes in NUDT5 inhibitor activity. We will therefore examine which substituents can boost the action of NUDT5 inhibitors.

## 2 Materials and methods

### 2.1 Protein structure preparation and docking validation

The target protein used in this computational study was NUDT5, a well-known target for breast cancer treatment. The X-ray diffraction structure of the NUDT5 breast cancer regulator (PDB code **5NWH**), which interacts with compound 7-[[5-(3, 4-dichlorophenyl)-1,3,4-oxadiazol-2-yl]methyl]-1,3-dimethyl-8-piperazin-1-yl-purine-2,6-dione (**9CH**), is available at https://www.rcsb.org/structure/5NWH. Its crystal structure has a resolution of 2.6 Å [10, 26]. Protein structure preparation was conducted using the BIOVIA Discovery Studio Visualizer v21.1.1.20298. Some of the steps for preparation included water removal, the addition of polar hydrogen, administration of Gasteiger charge, and separation of target proteins (NUDIX5) and the co-crystallized ligand (**9CH**) [33].

Docking was validated through AutoDock version 1.5.6 and was performed by redocking compound 7-[[5-(3, 4-dichlorophenyl)-1,3,4-oxadiazol-2-yl]methyl]-1,3-dimethyl-8-piperazin-1-yl-purine-2,6-dione (**9CH**) in a NUDT5 binding site. The docking parameter was considered valid if it had a root mean square deviation (RMSD) value of <2 Å [19]. The grid box used in the molecular docking was cubic, with coordinates x, y, z (40 × 40 × 40), 0.375 Å spacing, and grid centers x, y, z (12,396; -13,752; -13.546). The grid box files were stored as .gpf files, the autogrid procedure was performed using a command prompt, and the docking process was conducted on the compounds and proteins. The pdbqt file in the genetic algorithm (GA) parameter had the number of runs set as 100 (the other parameters were set up by default). The output file was stored with Lamarckian GA with the file type .dpf. The .dpf file was run through the command prompt. The parameters are based on the Amber force field. The binding affinity values, inhibition constants, and best conformation were obtained from the docking results [4, 25, 38].

### 2.2 Preparation of metal complex compounds

Nine thiourea-iron (III) metal complex compounds were designed, as shown in Figure 3. All complex compounds were interpreted using Marvin 21.12.0, ChemAxon (https://www.chemaxon.com, accessed on 14 July 2021). Some of the steps performed on the complex compounds were carried out after the complex compound was generated, protonated at pH 7.4, and stored as a .mrv file. Then, the conformation (energy optimization) with the force field parameter of MMFF94, with a maximum number of conformers 10, was determined on the .mrv type file, and the structure of the transformation that had the lowest energy was obtained from the conformation results and stored as a .mol2 type file.

AutoDock version 1.5.6 was used for the molecular docking of complex compounds against NUDT5 [3, 12, 20, 29]. All complex compound optimization results from the Marvin suite were interpreted .pdbqt files with AutoDock. Molecular docking of the complex compounds was carried out using the same parameters used during the docking validation process.

### 2.3 Analysis of docking results

The 2D/3D analysis and visualization of the docking results were performed using BIOVIA Discovery Studio Visualizer v21.1.1.20298 (https://discover.3ds.com/discovery-studio-visualizer-download, accessed on 24 July 2021). The analyses included the structure overlay of docking validation results and the types of interactions between the compounds and proteins.

### 2.4 Molecular dynamics simulation analysis

The molecular dynamics (MD) study was conducted on four of the best complex compounds obtained from the docking results and the comparison compound/native ligand (compound **10**; **9CH**) for 100 ns, using the Desmond software Release 2019-2 for academic licensing (Schrödinger, LLC, New York, NY, USA) to study the stability of interactions from the ligand-2NWH complex. The simulation used a TIP3P water model and 0.15 M NaCl to mimic a physiological ionic concentration. Energy minimization was performed for 100 ps. The MD simulation was run for 100 ns at 300 K and standard pressure (1.01325 bar), with an orthorhombic box with a dimension buffer of 10 Å × 10 Å × 10 Å and an NPT ensemble. The energy was recorded at intervals of 1.2 ps. The protein-ligand complex was neutralized by the addition of Na^+^ or Cl^−^ ions to balance the MD-simulated net charge system. The Nosé-Hoover chain and Martyna-Tobias-Klein algorithms were used to maintain the temperature of all MD systems at 300 K and the pressure at 1.01325 bar. All well-minimized and equilibrated systems were subjected to a 100-ns MD run in the NPT ensemble with periodic boundary conditions using the OPLS 2005 force field parameters [13, 17, 24, 37].

## 3 Results and discussion

### 3.1 Validation docking and docking analysis of the ligand–NUDT5 complexes

**Figure 4:**
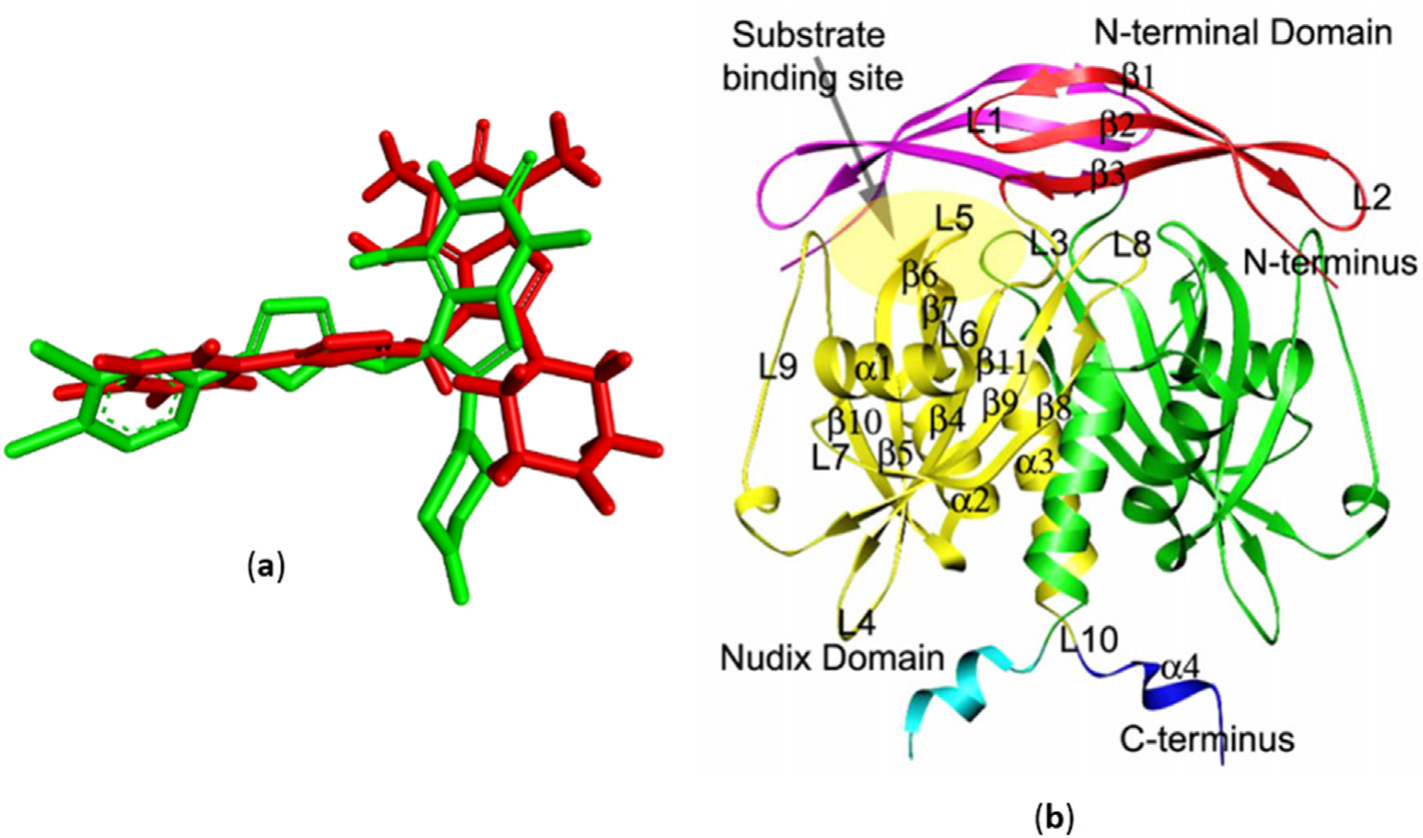
(a) Overlay of the **9CH** structure between the redocking result (green) and the crystal structure (red); (b) Structure of NUDT5 with substrate [45].

The results of the docking validation, which was carried out by redocking the native ligand (**9CH**) in the NUDT5 binding site, showed an RMSD value of 1.92 Å for each complex. The overlay structure from the redocking results with the crystal structure is shown in Figure 4(a), and the structure of NUDT5 [45] is shown in Figure 4(b).

As the RMSD value (1.92 Å) was <2 Å, it can be concluded that the method used is valid and valuable for docking studies of the designed complex compounds of Fe (III) metal.

Using AutoDock and the same parameters as in the docking validation procedure, the binding affinity (kcal/mol) and types of interactions in the ligand–NUDT5 complexes were obtained, as shown in Table 1.

**Table 1:** Binding affinity, inhibition constant, and interaction of each iron (III) complex.

No.	Binding affinity (kcal/mol)	Inhibition constant	Interaction residue		
			Conventional hydrogen bond	Carbon hydrogen bond	van der Waals bond
**1**	-5.88	48.91 mM	GluA:166, ArgA:84		GlyA:61, ValA:62, ArgA:51, GlyA:97, AspA:60, ProA:85, IleA:99, ThrA:53, ArgA:54, AspA:100, GluA:112, GluA:116, PheA:83, MetA:132, GluA:93
**2**	-8.77	375.06 nM	AspA:100, ArgA:111, LeuA:98	GluA:115, GluA:112, GlyA:97, GluA:166	MetA:132, CysA:139, ArgA:51, ThrA:53, GluA:116, ArgA:84, IleA:141
**3**	-7.88	1.69 mM	ThrA:53, AspA:100, ArgA:111, LeuA:98	GlyA:97, GluA:115	IleA:99, GluA:112, ArgA:51, IleA:141, MetA:132, AlaA:96, ArgA:84, GluA:166
**4**	-6.181	29.67 mM	GluA:166, LeuA:98	GlyA:97, GluA:112	SerA:137, AspA:133, GlyA:61, GlnA:82, GluA:116, ArgA:51
**5**	-8.57	525.17 nM	AspA:100, ArgA:111, LeuA:98	GluA:166, GluA:115	GluA:112, GlyA:61, ValA:62, AspA:60, ArgA:51, Arg:84, ThrA:53, ThrA:52, LysA:27
**6**	-9.56	98.54 nM	LeuA:98, AspA:100	GluA:112, AlaA:96, GlyA:97	GluA:116, GluA:115, ArgA:111, GluA:166, ArgA:84, ArgA:51, MetA:132, GlyA:61, Cys:139, IleA:141, ThrA:53
**7**	-9.25	165.04 nM	LeuA:98, GluA:112	TrpA:28, GluA:116, GlysA:97, GluA:115	ArgA:51, ArgA:84, ValA:158, GlnA:82, GluA:166, GlyA:61, ArgA:111
**8**	-8.11	1.14 mM	AspA:100, ArgA:111, LeuA:98	GluA:112, GluA:115, GluA:166	ThrA:53, GlyA:97, AlaA:96, ArgA:84, ArgA:51
**9**	-8.63	472.65 nM	AspA:100, LeuA:98, ArgA:111	GluA:166, GluA:115	Thr:53, ArgA:51, ArgA:84, AlaA:96, GluA:112
**10**	-6.08	34.71 mM	TrpA:46, AspA:133, ValA:49		MetA:132, SerA:137, SerA:48, GluA:47

Table 1 shows that except for compound 1, all the complex compounds have lower binding affinities than compound **10** (**9CH** as a co-crystallized ligand or comparison compound), suggesting that all ligand–NUDT5 complexes except compound 1 have a more stable interaction than compound **10**. Based on the binding affinities, the compound **6**–NUDT5 complex has the lowest energy (−9.56 kcal/mol) with an inhibition constant value of 98.54 nM, two conventional hydrogens (LeuA:98, AspA:100), three carbon-hydrogen (GluA:112, AlaA:96, GlyA:97), and 11 van der Waals (GluA:116, GluA:115, ArgA:111, GluA:166, ArgA:84, ArgA:51, MetA:132, GlyA:61, CysA:139, IleA:141, ThrA:53) bonds. The iron (III) metal complex structure and detailed docking results are shown in Supplementary Table S1. The conventional hydrogen bond with residue LeuA:98 occurred in eight complex compounds, including compound **2**; therefore, LeuA:98 possibly plays an important role in the stability of the interactions in the ligand–NUDT5 complex. The 2D/3D visualizations of compounds **2** and **6** in NUDT5 are shown in Figure S1.

As seen in Figure S1, the active site of NUDT5 is more lipophobic (blue); therefore, the iron (III) metal complexes that have more hydrophobic groups had less favoured interactions than the lipophobic compounds. From the docking results, we selected the four best ligand–NUDT5 complexes (compounds **2**, **6**, **7**, and **9**) and compound **10** (comparison compound) for subsequent MD studies to evaluate the stability of the interactions.

### 3.2 Molecular dynamics simulation analysis

**Figure 5:**
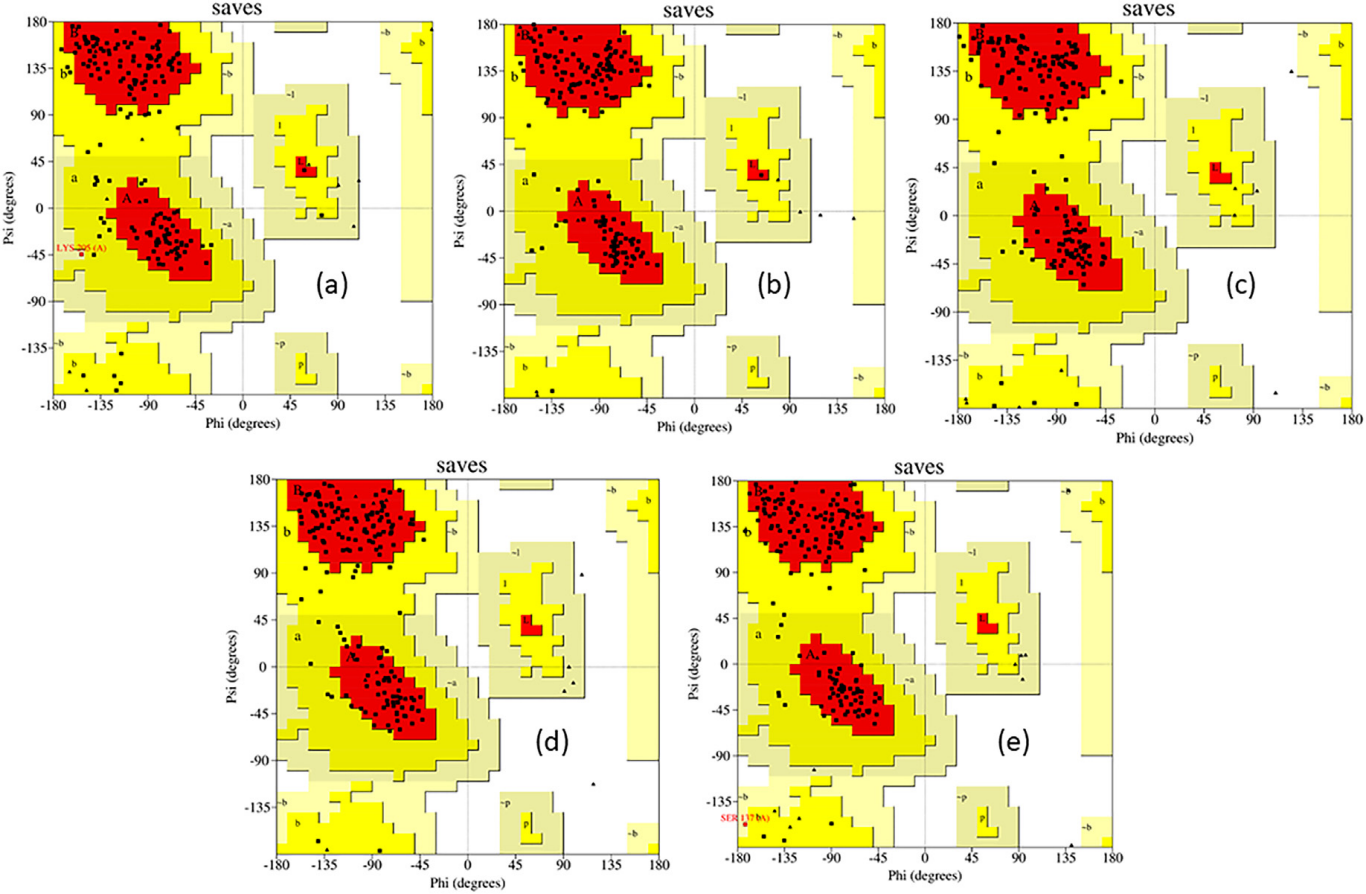
Ramachandran plots of ligand-NUDT5 results from 100 ns MD simulations: (a) compound **2**-NUDT5 complex, (b) compound **6**-NUDT5 complex, (c) compound **7**-NUDT5 complex, (d) compound **9**-NUDT5, and (e) compound **10**-NUDT5 complex.

MD simulations were performed for compound **2**-NUDT5, **6**-NUDT5, **7**-NUDT5, and **9**-NUDT5 complexes and compound **10**–NUDT5 (comparison compound). Some factors that influenced the results of MD simulations include compound conformation, water molecules, ions, cofactors, protonation of the compound, conformation, and solvent entropy [5, 9]. Several studies have reported the role and importance of MD simulations for MD refinement [17]. MD simulations were performed to determine the stability of the ligand-protein interactions from the docking results. The final structure of the MD simulation showed a strong stereochemical geometry of residues, consistent with the Ramachandran plot results shown in Figures 5a-5e.

In addition to the Ramachandran plots, the amount and percentage of residues in the favoured, allowed, and outlier regions of the five MD simulations performed are shown in Table 2.

**Table 2:** Amount and percentage of residue in favoured, allowed, and outlier regions of MD simulation.

System	Number and percentage of residues in favoured region	Number and percentage of residues in allowed region	Number and percentage of residues in the outlier region
Comp. 2-NUDT5 complex	137 (82.5%)	28 (16.9%)	1 (0.6%)
Comp. 6-NUDT5 complex	151 (91.0%)	15 (9.0%)	0 (0%)
Comp. 7-NUDT5 complex	145 (87.3%)	21 (12.7%)	0 (0%)
Comp. 9-NUDT5 complex	146 (88.0%)	20 (12.0%)	0 (0%)
Comp. 10-NUDT5 complex	146 (88.0%)	19 (11.4%)	1 (0.6%)

As evidenced in Figures 5a-5e and Table 2, compound **2**–NUDT5 and 10–NUDT5 complexes have residues present in the outlier region, as much as one residue. Compound 6/7/9–NUDT5 complexes are more attractive, as these had no residues present in the outlier region. At the end of the 100ns MD simulation, according to the Ramachandran plot, the protein-ligand system of the dynamic simulation was stable, as seen from the significant number of amino acids placed in the outlier region of less than 20% and the large number of amino acids located in the favoured region. Furthermore, the complex stability of all ligand–NUDT5 compounds during the 100-ns MD simulations was monitored in terms of the total amount of energy (E), potential energy (E_P), temperature (T), pressure (P), and volume (V) (Supplementary Figure S2–S6).

From the analysis of the 100-ns MD simulation quality parameters of all ligand–NUDT5 complexes, none of the 100-ns MD simulation quality parameters showed significant changes with respect to E, E_P, T, P, or V.

**Figure 6:**
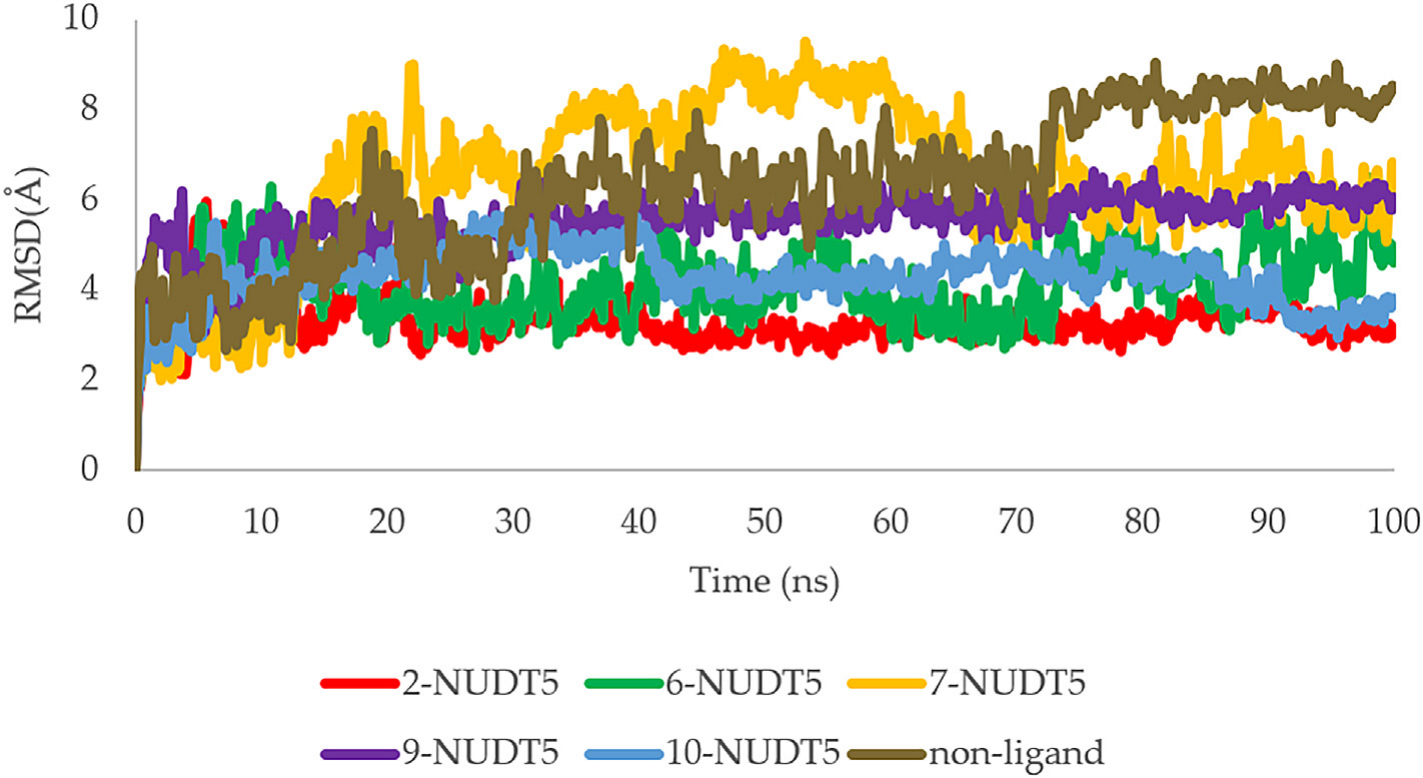
RMSD plots of ligand-NUDT5 complex: compound **2**-NUDT5 (red), compound **6**-NUDT5 (green), compound **7**-NUDT5 (orange), compound **9**-NUDT5 (blue), compound **10**-NUDT5 (purple), and NUDT5 non-ligand (brown).

In addition, the stability of the ligand–NUDT5 complex interaction is evident in the RMSD plot of the 100-ns MD simulation shown in Figure 6.

The significant changes in protein structure relative to the initial point can be detected using RMSD. The RMSD curve may flatten or level out, which is another sign that the protein has stabilized. As shown in the RMSD plot (Figure 6), compound **2**-NUDT5, **6**-NUDT5, **9**-NUDT5, and **10**-NUDT5 complexes had fluctuating RMSD values, starting from 0 to 3 ns and then stabilizing to 3 ns until the end of the 100-ns MD simulation. Although the compound **7**-NUDT5 complex shows a fluctuating RMSD ranging 0–65 ns, the value stabilizes at 65 ns until the end of the 100-ns MD simulation. At the end of the 100-ns MD simulation, all complexes had better interaction stability than the negative control (NUDT5 non-ligand). This is also evidenced by the large average, minimum, and maximum values of RMSD of NUDT5 non-ligand that were the largest as compared to all compound complexes. Given the Ramachandran plots and the percentage of residues in the favored regions, supported by the RMSD plots, it can be concluded that the complexes had interaction stability during the 100-ns MD simulations. The most stable interactions occurred with the compound **2**-NUDT5 complex, followed by compounds **6**-NUDT5, **10**-NUDT5, **9**-NUDT5, and **7**-NUDT5 complexes. These results are also supported by the magnitude of the average, minimum, and maximum RMSD values during the 100-ns MD simulation of each ligand–NUDT5 complex (Table 3).

**Table 3:** Average, minimum, and maximum of RMSD for ligand-NUDT5 complexes.

Complex	Average RMSD	Minimum RMSD	Maximum RMSD
Comp. 2-NUDT5 complex	3.304	1.233	5.951
Comp. 6-NUDT5 complex	4.089	1.876	6.476
Comp. 7-NUDT5 complex	6.421	1.838	9.507
Comp. 9-NUDT5 complex	5.515	2.164	6.640
Comp. 10-NUDT5 complex	4.285	1.457	5.633
NUDT5 non-ligand	6.329	1.944	9.034

The RMSD ligands of compounds **2**, **7**, and **10**-NUDT are shown in Figure S7.a. The root mean square fluctuation (RMSF) proteins were monitored to determine the flexibility of local residues, and RMSF ligands were evaluated to see ligand-wise atomic fluctuations. With fluctuations of dynamic systems above the well-determined average, the RMSD of the average over time can be referred to as the RMSF [17]. One of the RMSF ligand displays of compound **6** is shown in Figure S7.b.

**Figure 7:**
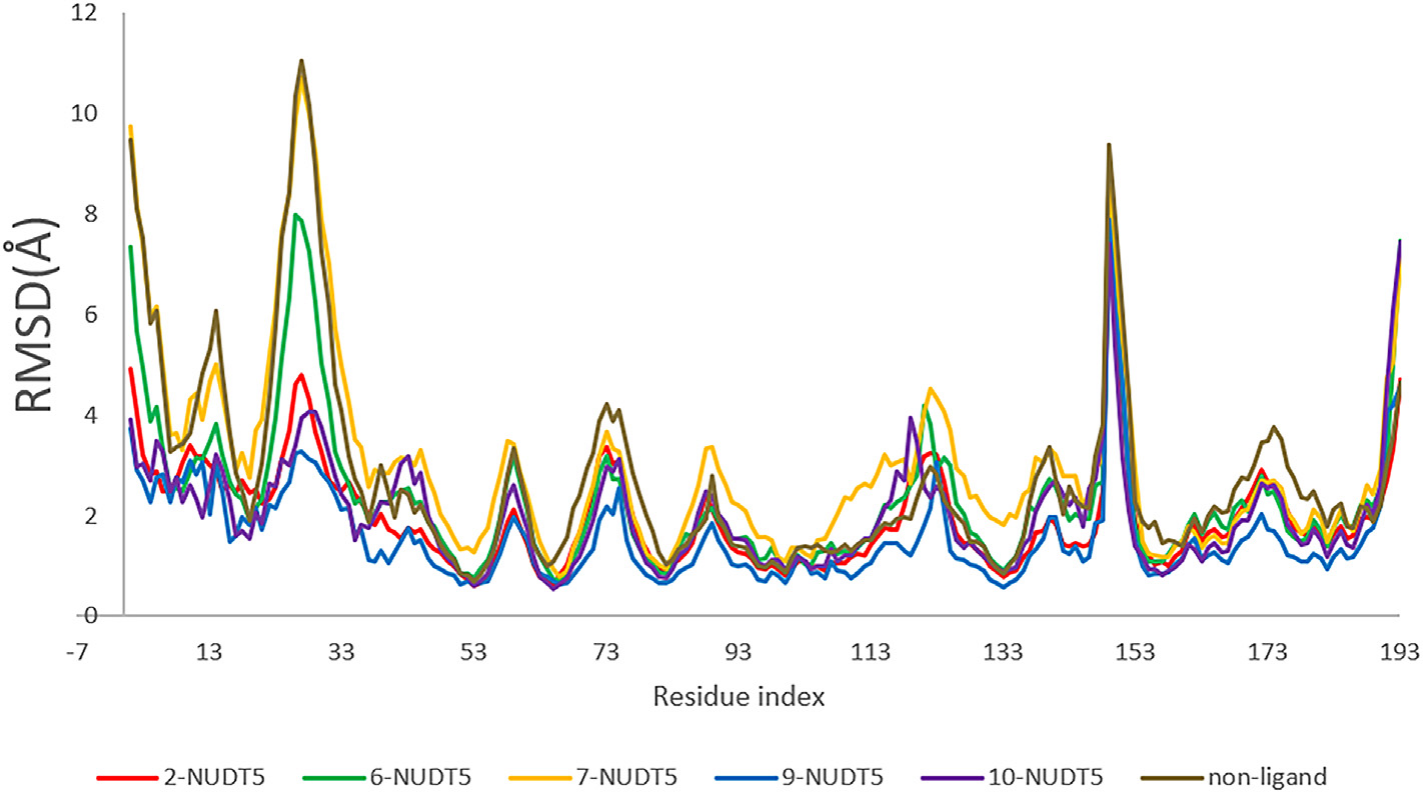
RMSF graph of complex systems of compound **2**-NUDT5, **6**-NUDT5, compound **7**-NUDT5, **9**-NUDT5, and compound **10**-NUDT5 complexes and NUDT5 non-ligand.

The RMSF graph for ligand–NUDT5 complexes can be used to evaluate the stability of each interaction. For example, Figure 7 shows the RMSF graph of compound **2**-NUDT5, **6**-NUDT5, **7**-NUDT5, **9**-NUDT5, and **10**-NUDT5 complexes during a 100-ns MD simulation. The residues show fluctuations in the same area, and the compound **9**-NUDT5 complex has the lowest fluctuation (blue). The average RMSF of the following compound–ligand complexes are as follows: **2**-NUDT5 (1.968 Å), **6**-NUDT5 (2.323 Å), **7**-NUDT5 (3.019 Å), **9**-NUDT5 (1.587 Å), and **10**-NUDT5 (2.006 Å). The fact that the residues forming the helix and sheet conformations have smaller RMSF values than the loop regions shows that the protein is stiffer due to the secondary structures.

The RMSF graph analysis can also identify the amino acid residues that contact/interact with the ligand, as seen in Figure 7, which shows the RMSF fluctuations. The contact residues with compound **6** and other complexes are shown in Figures S8–S11.

**Figure 8:**
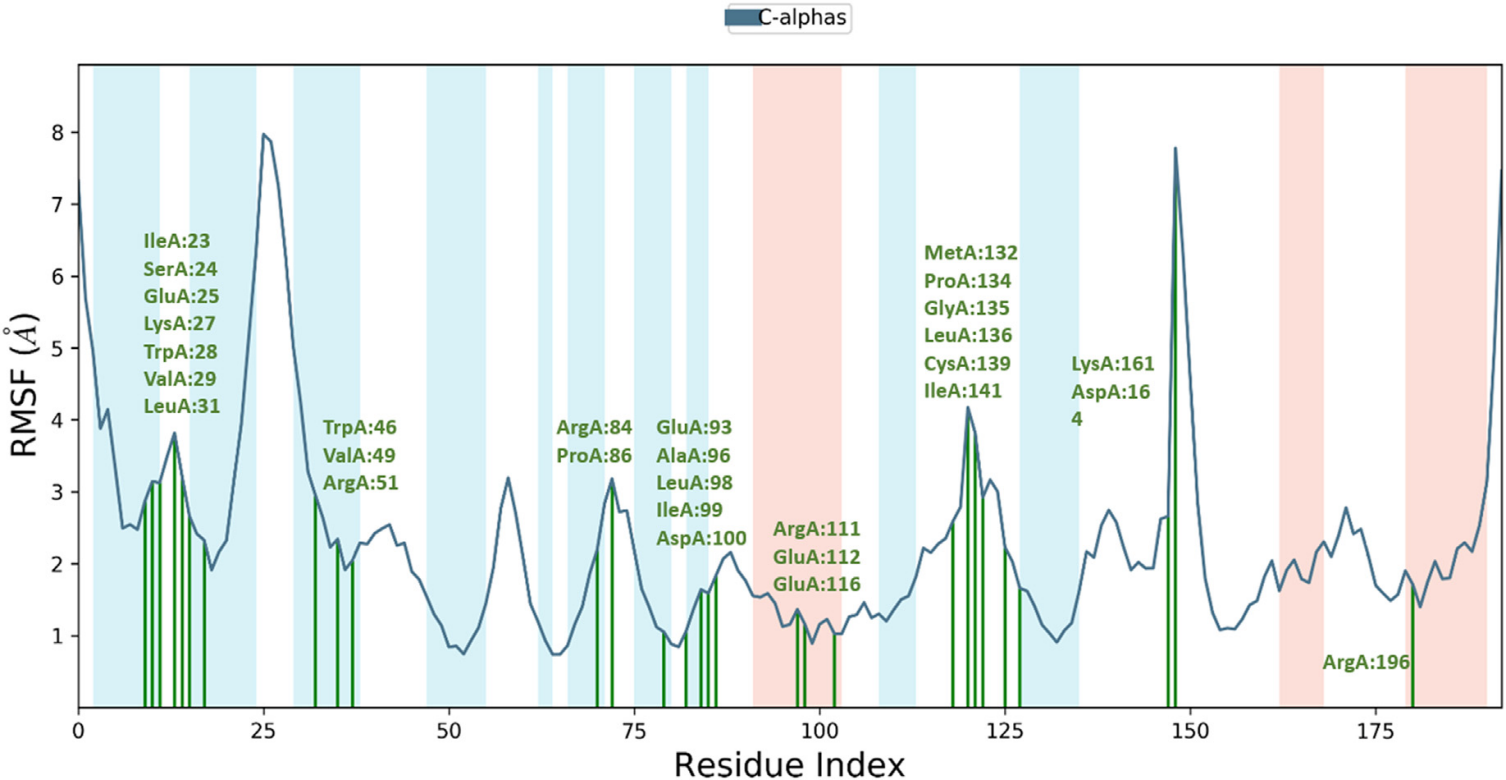
RMSF graph and residue contacts on the compound **6**-NUDT5 complex in 100 ns MD simulations.

**Figure 9:**
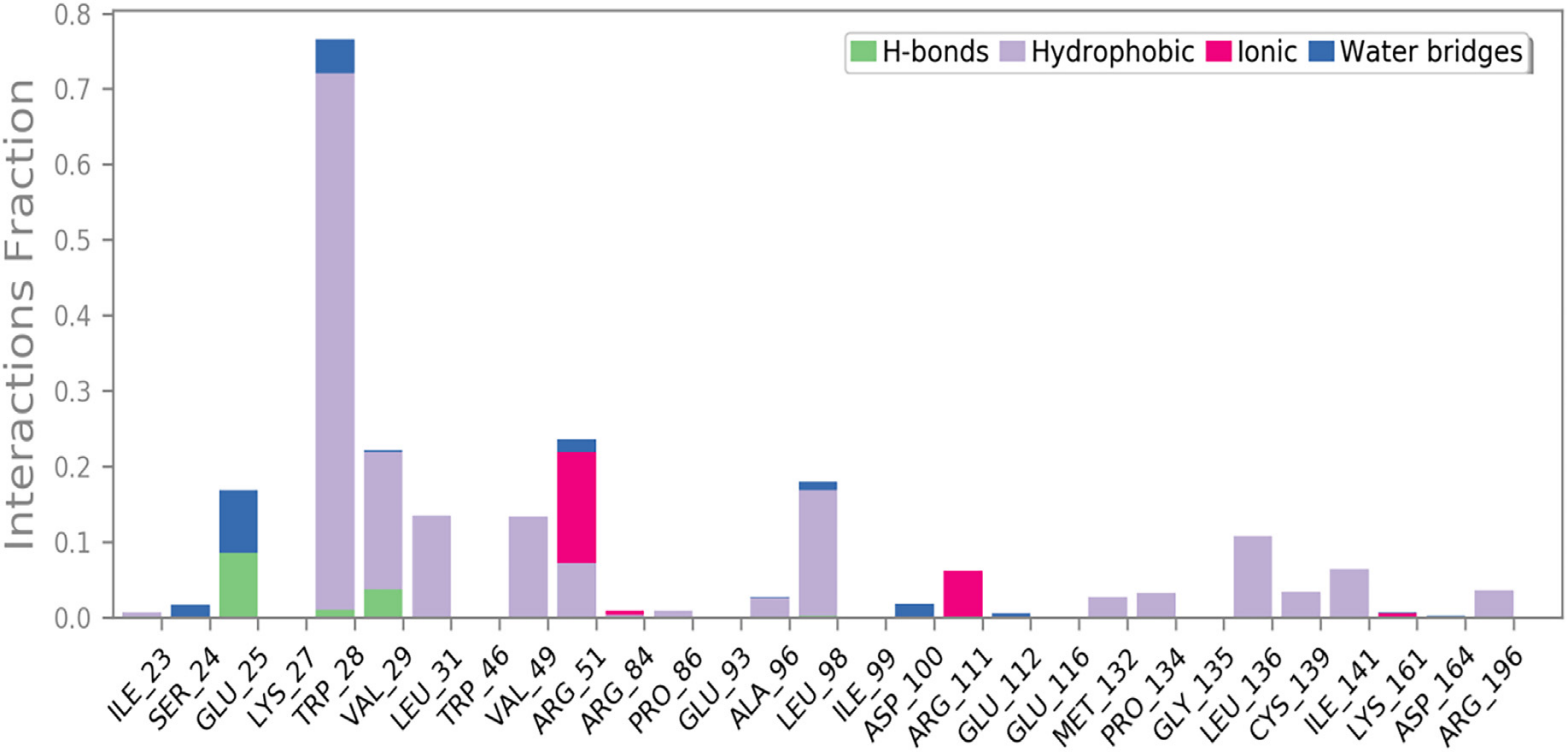
The histogram of the contact residues in compound **6**-NUDT5 complex.

As shown in Figure 8 and Figure 9, the 29 contact residues with compound **6**. These residues interacted through hydrogen bonds (Glu:25, Trp:28, Val:29), hydrophobic (TrpA:28, ValA:29, LeuA:31, ValA:49, ArgA:51, ProA:86, AlaA:96, LeuA:98, MetA:132, ProA:134, LeuA:136, CysA:139, IleA:141, and ArgA:196), ionic (ArgA:51, ArgA:84, ArgA:111) and water bridges (IleA:23, GluA:25, ArgA:51, LeuA:98, AspA:100, GluA:112) (for other complexes, see Figure S12–S15 and S16–S19).

Analysing the contact residues of the compound **2**-NUDT5 complex revealed 19 contact residues (LysA:27, TrpA:28, ValA:29, ValA:49, ArgA:51, GluA:82, ArgA:84, GluA:93, AlaA:96, ArgA:111, GluA:112, GluA:115, GluA:116, MetA:132, CysA:139, IleA:141, CysA:161, AspA:164, and GluA:166); for the compound **10**-NUDT5 complex, 27 residues (GluA:25, LysA:27, TrpA:28, ArgA:51, ThrA:52, ThrA:53, ArgA:54, LysA:55, GluA:56, GlnA:82, PheA:83, ArgA:84, ProA:85, ProA:86, MetA:87, GluA:93, AlaA:96, LeuA:98, GluA:112, GluA:115, GluA:116, MetA:132, LysA:161, AspA:164, GluA:166, AspA:194, and ArgA:196) were identified.

Figure S20 shows that the residue interacts with the ligands in each trajectory frame. Some protein residues show more than one specific contact with ligands, characterized by a darker orange color. Overall, six parameters [17] were analysed to explain the stability of compound **6** in NUDT5 in 100-ns MD simulations, as presented in Figure S21 (for other complexes, see Figure S22–S25).

Figure S21 shows the fluctuating RMSD ligands during the simulation process of compound 6. Initially, fluctuations were observed from 0 to 60 ns; a constant RMSD value was then observed throughout the simulation process. Fluctuations in the gyration radius were recorded up to 50 ns, and a stable conformation was obtained during a complete simulation of the process. The gyration radius of compound **6** in the 100-ns MD simulation ranged from 4.167 to 4.777 **Å**. The solvent accessible surface area (SASA) plot revealed fluctuating patterns to 60 ns that became stable until the simulation was complete. The molecular surface area (MolSA) plot suggests the stability of compound **6** during the simulation process. In contrast, the polar surface area (PSA) plot shows fluctuated RMSD to 42 ns that stabilized until the end of the 100-ns simulation. Moreover, intramolecular hydrogen bonding was not observed for compound **6** throughout the simulation process.

**Figure 10:**
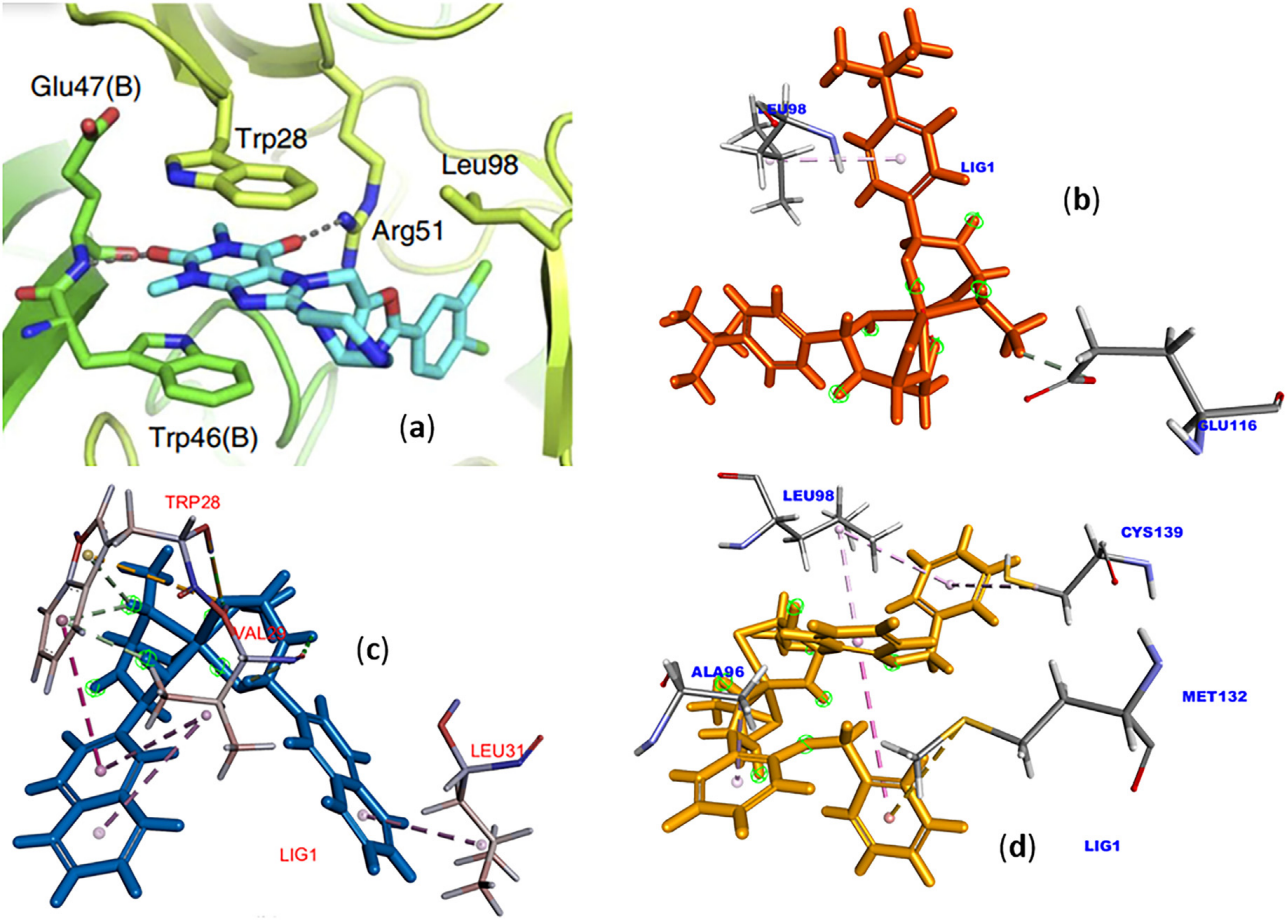
(a) The complex of NUDT5 with TH5427 [26], (b) Structure of 2-NUDT5. (c) 6-NUDT5, and (d) 7-NUDT5 complex.

Other studies have also reported that TrpA:28, TrpA:46, GluA:47, ArgA:51, and LeuA:98 residues affect the stability of interactions in the ligand–NUDT5 complex [26], as shown in Figure 10 (a). From the interactions in Figure 10(b)-10(d), it can be seen that the 6-NUDT5 complex has a more harmonious interaction than the others, among others, through interaction with TrpA:28 through hydrogen and hydrophobic interactions.

**Figure 11:**
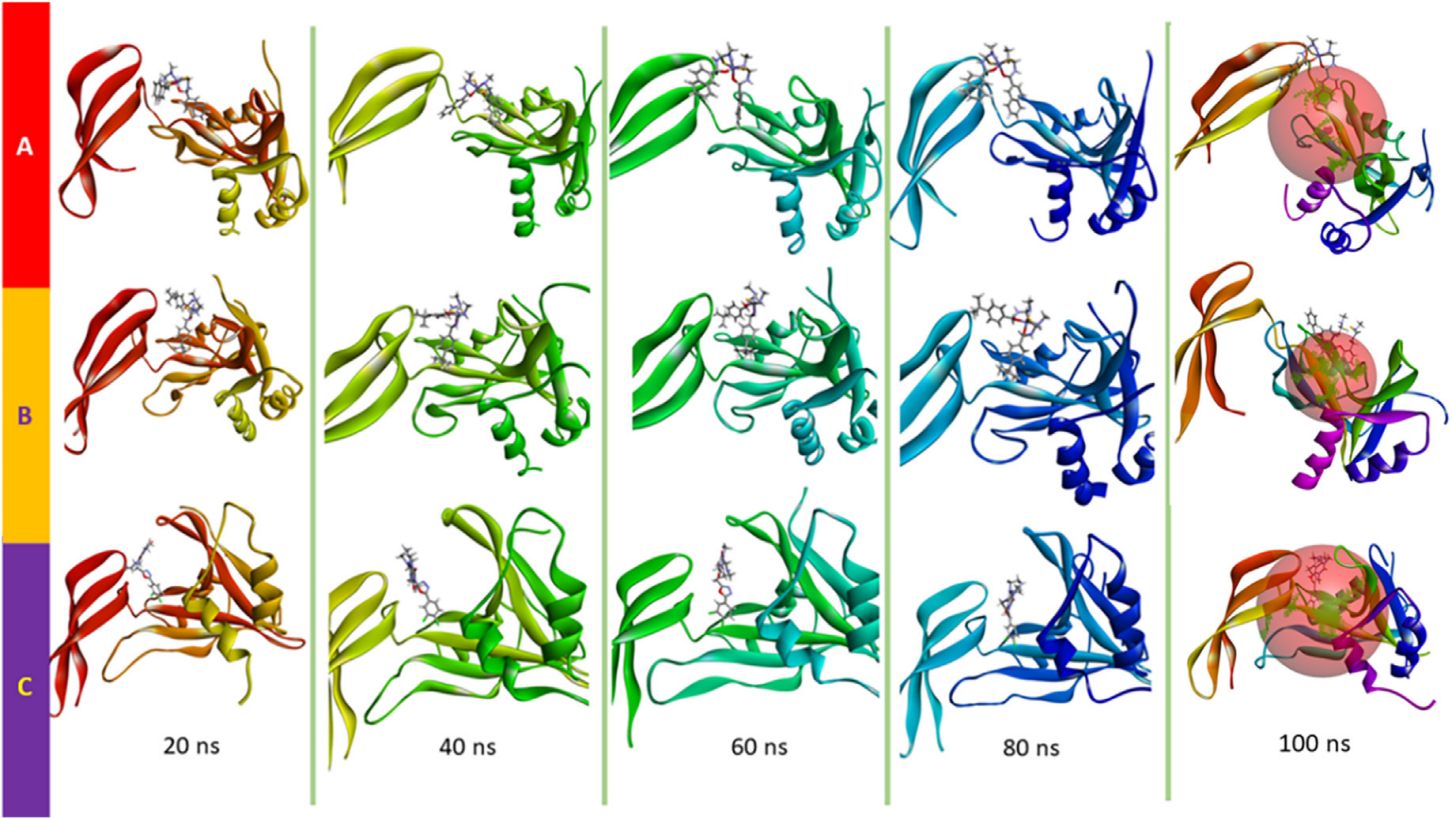
The trajectory conformation changes of compound **6**-NUDT5 (A), compound **7**-NUDT5 (B), and compound **10**-NUDT5 complexes (C) in 100 ns MD simulations.

Changes in the conformation of the ligand–NUDT5 complex were also observed in the MD simulation results. Figure 11(A)–(C) shows the conformational changes observed in the trajectory during the 100-ns MD simulation, ranging from 20, 40, 60, 80, and 100 ns.

In three of the complexes (compounds **6**-NUDT5, **7**-NUDT5, and **10**-NUDT5), conformational changes ranged from the beginning till the end of the 100-ns MD simulation. However, the conformational changes were still in the binding site of the NUDT5 protein (Figure 11).

In Figure 10(b)–(d), it can be seen that differences in metal complex compounds can lead to differences in amino acids that bind to each ligand. However, despite the changes in the metal complex structure, after the MD process was completed, the metal complex compounds still interacted around the binding site as seen in Figure 11(A)–(C), so they are predicted to have similar interaction with NUDT5 in its anticancer activity.

## 4 Conclusions

Based on the molecular docking results for NUDT5, it can be concluded that all iron (III) metal complexes (compounds **2**, **3**, **4**, **5**, **6**, **7**, **8**, and **9**), except for compound **1,** have lower binding affinities than the compound **10** (**9CH**).

Evaluating the interaction stability results from the molecular docking through the 100-ns MD simulation revealed that compounds **2** and **6** had better interaction stability than other compounds had. They had the lowest RMSD and RMSF values compared with the other compounds. Therefore, further research could be carried out on these two compounds in the discovery and development of new compounds as drug candidates for breast cancer treatment.

## Declarations

### Author contribution statement

Ruswanto Ruswanto: Performed the experiments; Analyzed and interpreted the data; Wrote the paper.

Tita Nofianti, Richa Mardianingrum: Performed the experiments.

Dini Kesuma: Contributed reagents, materials, analysis tools or data.

Siswandono: Conceived and designed the experiments; Wrote the paper.

### Funding statement

This work was supported by Fundamental Research Grant (No. Contract: 19/SP2H/RDPKR-JAMAK/LL4/2021), Ministry of Education, Culture, Research, and Technology of Indonesia in 2021.

### Data availability statement

Data included in article/supplementary material/referenced in article.

### Declaration of interests statement

The authors declare no conflict of interest.

### Additional information

No additional information is available for this paper.

## Appendix A Supplementary data

suplemantary final2 suplemantary final2
