# Corrigendum to “Design, molecular docking, and molecular dynamics of thiourea-iron (III) metal complexes as NUDT5 inhibitors for breast cancer treatment” [Heliyon 8 (9) (2022) e10694]

**DOI:** 10.1016/j.heliyon.2022.e11273

**Published:** 2022-11-02

**Authors:** Ruswanto Ruswanto, Tita Nofianti, Richa Mardianingrum, Dini Kesuma, Siswandono

**Affiliations:** a Faculty of Pharmacy, Universitas Bakti Tunas Husada, Tasikmalaya, Indonesia; b Department of Pharmacy, Universitas Perjuangan, Tasikmalaya, Indonesia; c Faculty of Pharmacy, Universitas Surabaya, Surabaya, Indonesia; d Faculty of Pharmacy, Universitas Airlangga, Surabaya, Indonesia

In the original published version of this article, Figure 1 and its corresponding caption had been reused without reference to the original source. The corrected version of the caption for Figure 1 can be found below. The authors apologize for the error. Both the HTML and PDF versions of the article have been updated to correct the errors.

Figure 1. The role of NUDT5 in breast cancer metastasis. Model showing the multiple roles and indications for a key role of NUDT5 in aggressive breast cancer. (1) NUDT5 is elevated in tumor versus normal breast cancer tissue. (2) NUDT5 is essential for breast cancer stem cell (BCSC) generation and maintenance. (3) NUDT5 is highly expressed in circulating tumor cells (CTCs). (4 and 5) Elevated levels of NUDT5 are associated with increased levels of recurrence and metastasis in patients suggesting a role in mesenchyme to epithelial transition and secondary site colonization. And finally, (6) analysis of the gene expression changes occurring in BCSC in 3D cell culture suggests a role of NUDT5 in angiogenesis. (Figure and figure caption taken from Wright, R.H.G.; Beato, M. Role of the NUDT Enzymes in Breast Cancer. Int. J. Mol. Sci. 2021, 22, 2267. https://doi.org/10.3390/ijms22052267).

## Declaration of interests statement

The authors declare no conflict of interest.

