# Supplementary material for: Design, molecular docking, and molecular dynamics of thiourea-iron (III) metal complexes as NUDT5 inhibitors for breast cancer treatment
Source: Heliyon. 2022 Sep 19;8(9):e10694. doi: 10.1016/j.heliyon.2022.e10694 (PMC9513778; doi:10.1016/j.heliyon.2022.e10694)
Supplement: suplemantary final2 [file mmc1.docx]

Supplementary Material

**Design, Molecular Docking, and Molecular Dynamics of Thiourea-Iron (III) Metal Complexes as NUDT5 Inhibitors for Breast Cancer Treatment**

**List of the Contents**

**Table S1**: The docking result of the whole ligand-NUDT5 complex,

**Fig. S1.** Two-/three-dimensional visualization of molecular docking results: (a) compound 6-NUDT5 complex and (b) compound 2-NUDT5 complex,

**Fig. S2.** Molecular dynamics simulation quality parameters for the compound 2-NUDT5 complex for 100 ns

**Fig. S3.** The Molecular dynamic simulation quality parameters for the compound **6**-NUDT5 complex for 100 ns,

**Fig. S4.** The Molecular dynamic simulation quality parameters for the compound **7**-NUDT5 complex for 100 ns,

**Fig. S5.** The Molecular dynamic simulation quality parameters for the compound **9**-NUDT5 complex for 100 ns,

**Fig. S6.** The Molecular dynamic simulation quality parameters for the compound **10**-NUDT5 complex for 100 ns,

**Fig. S7**. (a) RMSD ligands of compounds **2** (red), **6** (green), **7** (orange), **9** (blue), and **10** (purple); (b) RMSF ligands of compound **2**

**Fig. S8. The** RMSF graph and residue contacts on the compound 2-NUDT5 complex in 100 ns MD simulation,

**Fig. S9. The** RMSF graph and residue contacts on the compound 7-NUDT5 complex in 100 ns MD simulation,

**Fig. S10. The** RMSF graph and residue contacts on the compound 9-NUDT5 complex in 100 ns MD simulation,

**Fig. S11. The** RMSF graph and residue contacts on the compound 10-NUDT5 complex in 100 ns MD simulation,

**Fig. S12.** The histogram bar chart showing the contact residues in compound 2-NUDT5,

**Fig. S13.** The histogram bar chart showing the contact residues in compound 7-NUDT5,

**Fig. S14.** The histogram bar chart showing the contact residues in compound 9-NUDT5,

**Fig. S15.** The histogram bar chart showing the contact residues in compound 10-NUDT5, **Fig. S16.** Timeline representation of residue contacts and interaction of compound 2-NUDT5 in 100 ns MD simulation,

**Fig. S17.** Timeline representation of residue contacts and interaction of compound 7-NUDT5 in 100 ns MD simulation,

**Fig. S18.** Timeline representation of residue contacts and interaction of compound 9-NUDT5 in 100 ns MD simulation,

**Fig. S19.** Timeline representation of residue contacts and interaction of compound 10- NUDT5 in 100 ns MD simulation,

**Fig. S20**. Timeline representation of residue contacts and interaction of compound **6**-NUDT5 in 100 ns MD simulations,

**Fig. S21**. Ligand properties during 100 ns simulations for compound **6**: (A) ligand RMSD (root mean square deviation), (B) radius of gyration (rGyr), (C) NS34, (D) molecular surface area (MolSA), (E) solvent accessible surface area (SASA), and (F) polar surface area (PSA),

**Fig. S22**. Ligand properties during 100 ns simulations for compound 2: (A) ligand RMSD, root mean square deviation, (B) radius of gyration (rGyr), (C) NS34, (D) molecular surface area (MolSA), (E) solvent accessible surface area (SASA), and (F) polar surface area (PSA). **Fig. S23**. Ligand properties during 100 ns simulations for compound 7: (A) ligand RMSD, root mean square deviation, (B) radius of gyration (rGyr), (C) NS34, (D) molecular surface area (MolSA), (E) solvent accessible surface area (SASA), and (F) polar surface area (PSA), **Fig. S24.** Ligand properties during 100 ns simulations for compound 9: (A) ligand RMSD, root mean square deviation, (B) radius of gyration (rGyr), (C) NS34, (D) molecular surface area (MolSA), (E) solvent accessible surface area (SASA), and (F) polar surface area (PSA), **Fig. S25**. Ligand properties during 100 ns simulations for compound 10: (A) ligand RMSD, root mean square deviation, (B) radius of gyration (rGyr), (C) NS34, (D) molecular surface area (MolSA), (E) solvent accessible surface area (SASA), and (F) polar surface area (PSA).

**Table S1**. The docking result of the whole ligand-NUDT5 complex

| **No. of Complex** | **Conventional hydrogen**  **bond** | **Carbon hydrogen**  **bond** | **Van der Waals** | **Alkyl/ pi- alkyl** | **Pi-Pi T Shaped** | **Amide Pi- Stacked/Pi-**  **Pi Stacked** | **Unfavourable bump** | **Halogen** | **Covalent bond** | **Pi-sigma** | **Pi-Lone Pair** |
| --- | --- | --- | --- | --- | --- | --- | --- | --- | --- | --- | --- |
| **1** | GluA:166, |  | GlyA:61, | CysA:139, |  |  |  |  |  |  |  |
|  | ArgA:84 |  | ValA:62, | AlaA:96, |  |  |  |  |  |  |  |
|  |  |  | ArgA:51, | IleA:141, |  |  |  |  |  |  |  |
|  |  |  | GlyA:97, | ProA:86, |  |  |  |  |  |  |  |
|  |  |  | AspA:60, | LeuA:98, |  |  |  |  |  |  |  |
|  |  |  | ProA:85, | TrpA:28 |  |  |  |  |  |  |  |
|  |  |  | IleA:99, |  |  |  |  |  |  |  |  |
|  |  |  | ThrA:53, |  |  |  |  |  |  |  |  |
|  |  |  | ArgA:54, |  |  |  |  |  |  |  |  |
|  |  |  | AspA:100, |  |  |  |  |  |  |  |  |
|  |  |  | GluA:112, |  |  |  |  |  |  |  |  |
|  |  |  | GluA:116, |  |  |  |  |  |  |  |  |
|  |  |  | PheA:83, |  |  |  |  |  |  |  |  |
|  |  |  | MetA:132, |  |  |  |  |  |  |  |  |
|  |  |  | GluA:93 |  |  |  |  |  |  |  |  |
| **2** | AspA:100, | GluA:115, | MetA:132, | AlaA:96 | TrpA:28 |  |  |  |  |  |  |
|  | ArgA:111, | GluA:112, | CysA:139, |  |  |  |  |  |  |  |  |
|  | LeuA:98 | GlyA:97, | ArgA:51, |  |  |  |  |  |  |  |  |
|  |  | GluA:166 | ThrA:53, |  |  |  |  |  |  |  |  |
|  |  |  | GluA:116, |  |  |  |  |  |  |  |  |
|  |  |  | ArgA:84, |  |  |  |  |  |  |  |  |
|  |  |  | IleA:141 |  |  |  |  |  |  |  |  |
| **3** | ThrA:53, | GlyA:97, | IleA:99, | TrpA:28 |  |  |  |  |  |  |  |
|  | AspA:100, | GluA:115 | GluA:112, |  |  |  |  |  |  |  |  |
|  | ArgA:111, |  | ArgA:51, |  |  |  |  |  |  |  |  |
|  | LeuA:98 |  | IleA:141, |  |  |  |  |  |  |  |  |
|  |  |  | MetA:132, |  |  |  |  |  |  |  |  |
|  |  |  | AlaA:96, |  |  |  |  |  |  |  |  |

|  |  |  | ArgA:84,  GluA:166 |  |  |  |  |  |  |  |  |
| --- | --- | --- | --- | --- | --- | --- | --- | --- | --- | --- | --- |
| **4** | GluA:166, LeuA:98 | GlyA:97, GluA:112 | SerA:137, AspA:133, GlyA:61, GlnA:82, GluA:116,  ArgA:51 | IleA:141, CysA:139, MetA:132, ValA:29 |  | AlaA:96, TrpA:28 | ArgA:84 |  |  |  |  |
| **5** | AspA:100, ArgA:111, LeuA:98 | GluA:166, GluA:115 | GluA:112, GlyA:61, ValA:62, AspA:60, ArgA:51, Arg:84, ThrA:53, ThrA:52,  LysA:27 | MetA:132, AlaA:96, CysA:119, IleA:141 |  |  |  |  |  | GlyA:97, TrpA:28 |  |
| **6** | LeuA:98, | GluA:112, | GluA:116, |  | TrpA:28 |  |  |  |  |  |  |
|  | AspA:100 | AlaA:96, | GluA:115, |  |  |  |  |  |  |  |  |
|  |  | GlyA:97 | ArgA:111, |  |  |  |  |  |  |  |  |
|  |  |  | GluA:166, |  |  |  |  |  |  |  |  |
|  |  |  | ArgA:84, |  |  |  |  |  |  |  |  |
|  |  |  | ArgA:51, |  |  |  |  |  |  |  |  |
|  |  |  | MetA:132, |  |  |  |  |  |  |  |  |
|  |  |  | GlyA:61, |  |  |  |  |  |  |  |  |
|  |  |  | Cys:139, |  |  |  |  |  |  |  |  |
|  |  |  | IleA:141, |  |  |  |  |  |  |  |  |
|  |  |  | ThrA:53 |  |  |  |  |  |  |  |  |
| **7** | LeuA:98, | TrpA:28, | ArgA:51, |  |  |  |  |  |  |  | AlaA:96 |
|  | GluA:112 | GluA:116, | ArgA:84, |  |  |  |  |  |  |  |  |
|  |  | GlysA:97, | ValA:158, |  |  |  |  |  |  |  |  |
|  |  | GluA:115 | GlnA:82, |  |  |  |  |  |  |  |  |
|  |  |  | GluA:166, |  |  |  |  |  |  |  |  |

|  |  |  | GlyA:61,  ArgA:111 |  |  |  |  |  |  |  |  |
| --- | --- | --- | --- | --- | --- | --- | --- | --- | --- | --- | --- |
| **8** | AspA:100, ArgA:111, LeuA:98 | GluA:112, GluA:115, GluA:166 | ThrA:53, GlyA:97, AlaA:96, ArgA:84,  ArgA:51 |  | TrpA:28 |  |  | IleA:99 |  |  |  |
| **9** | AspA:100, LeuA:98, ArgA:111 | GluA:166, GluA:115 | Thr:53, ArgA:51, ArgA:84, AlaA:96,  GluA:112 |  | TrpA:28 |  |  |  |  | GlyA:97 |  |
| **10** | TrpA:46, AspA:133, ValA:49 |  | MetA:132, SerA:137, SerA:48,  GluA:47 | CysA:131 |  |  |  |  | SerA:137, AspA:133 |  |  |

**Fig. S1.** Two-/three-dimensional visualization of molecular docking results: (a) compound 6-NUDT5 complex and (b) compound 2-NUDT5 complex,


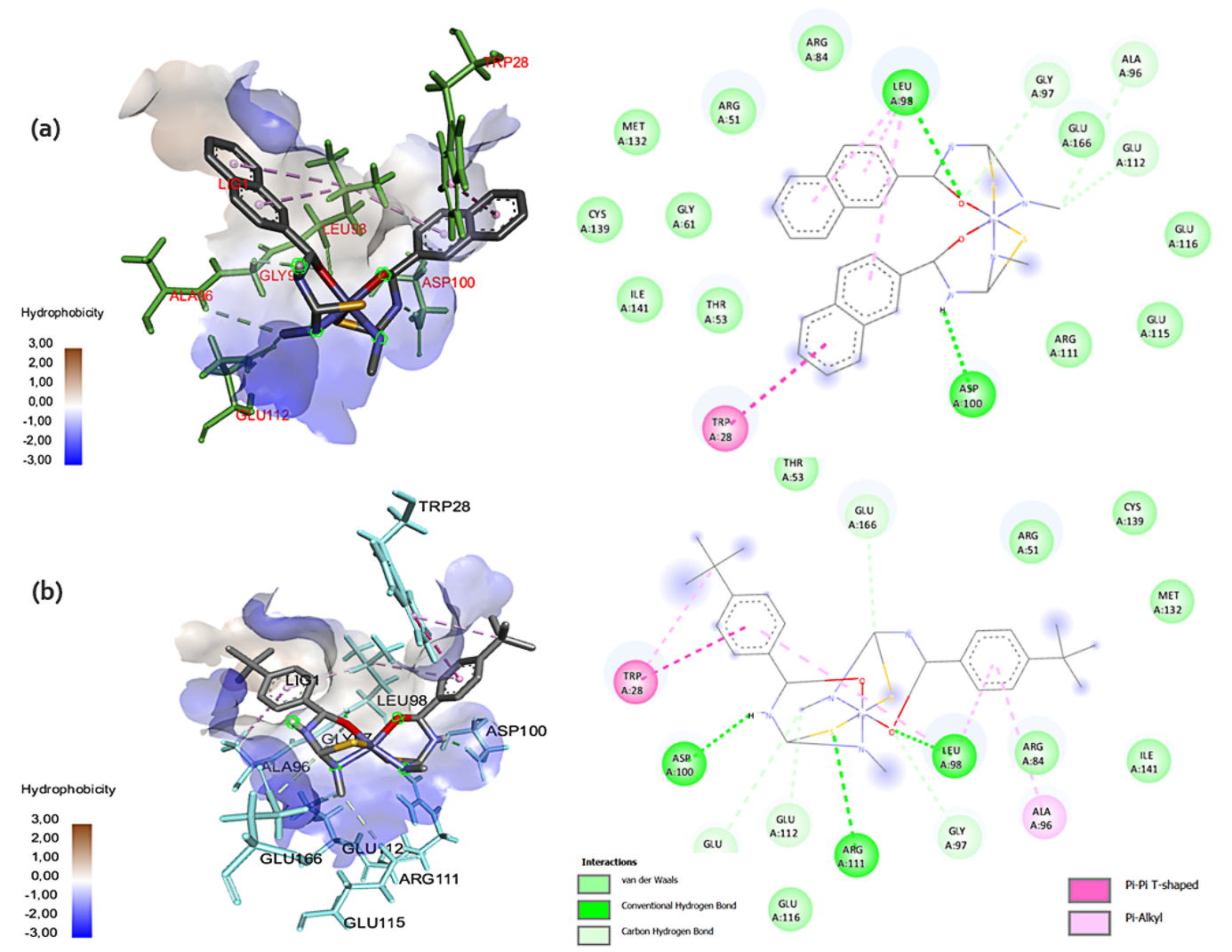


**Fig. S2.** Molecular dynamics simulation quality parameters for the compound 2-NUDT5 complex for 100 ns


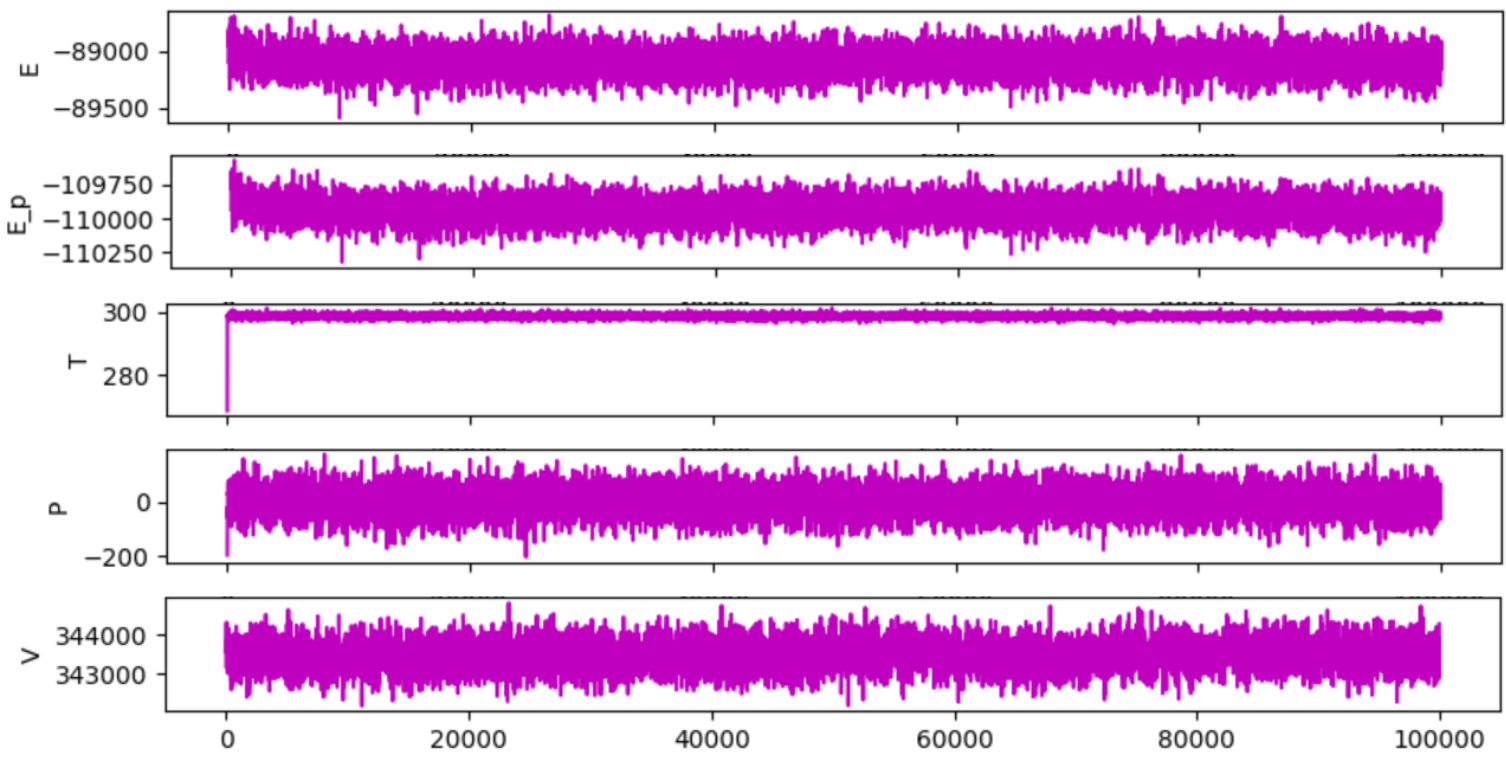


**Fig. S3.** The Molecular dynamic simulation quality parameters for the compound **6**-NUDT5 complex for 100 ns


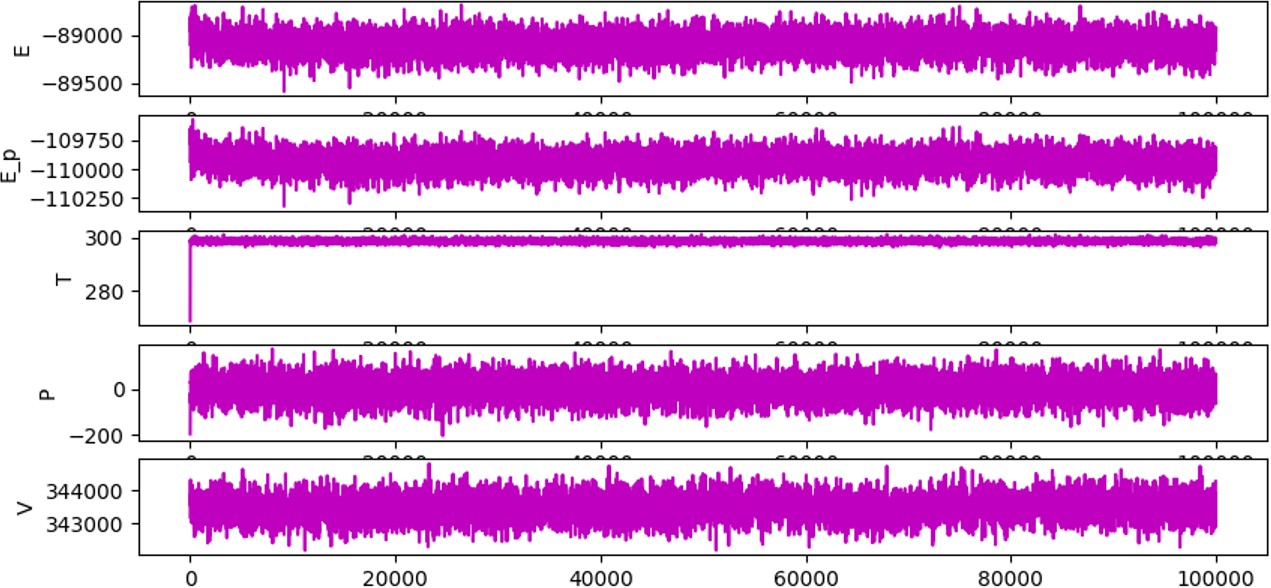


**Fig. S4.** The Molecular dynamic simulation quality parameters for the compound **7**-NUDT5 complex for 100 ns


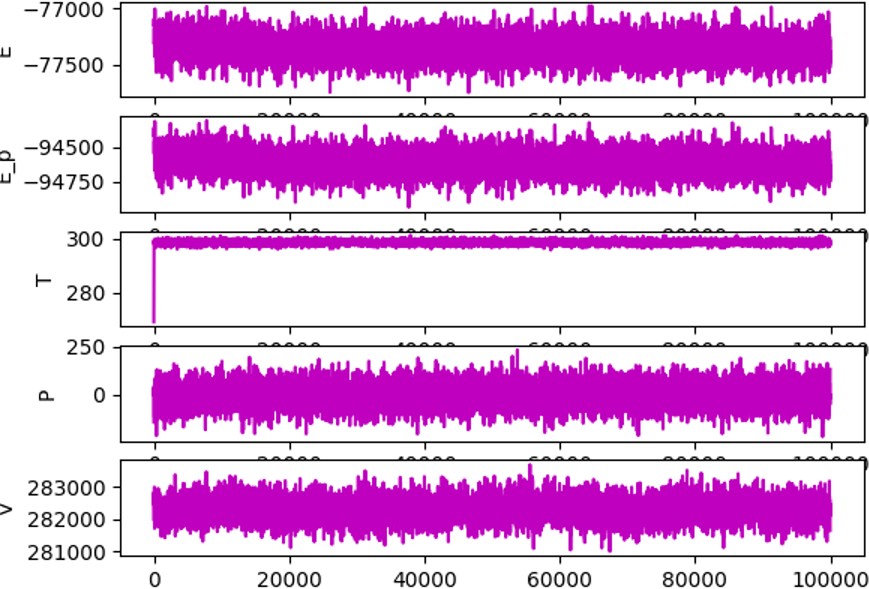


**Fig. S5.** The Molecular dynamic simulation quality parameters for the compound **9**-NUDT5 complex for 100 ns


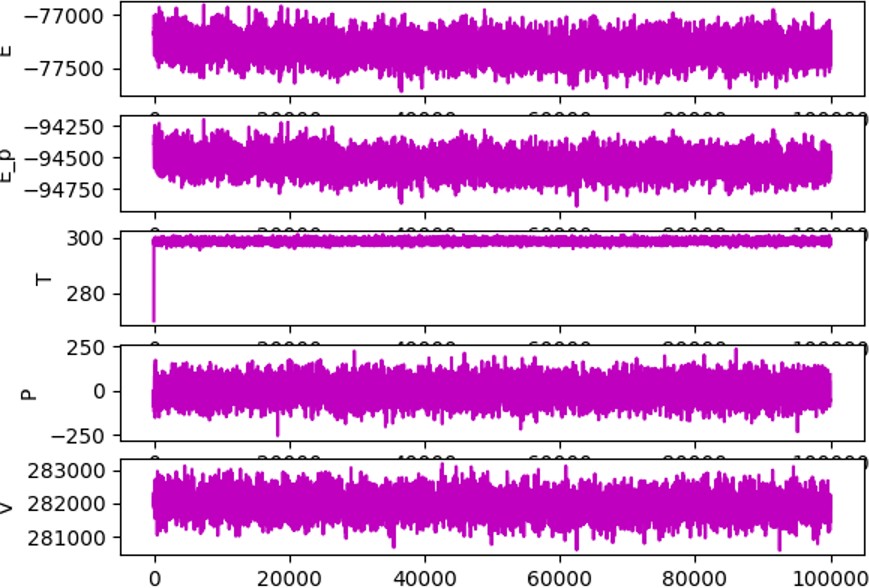


**Fig. S6.** The Molecular dynamic simulation quality parameters for the compound **10**-NUDT5 complex for 100 ns


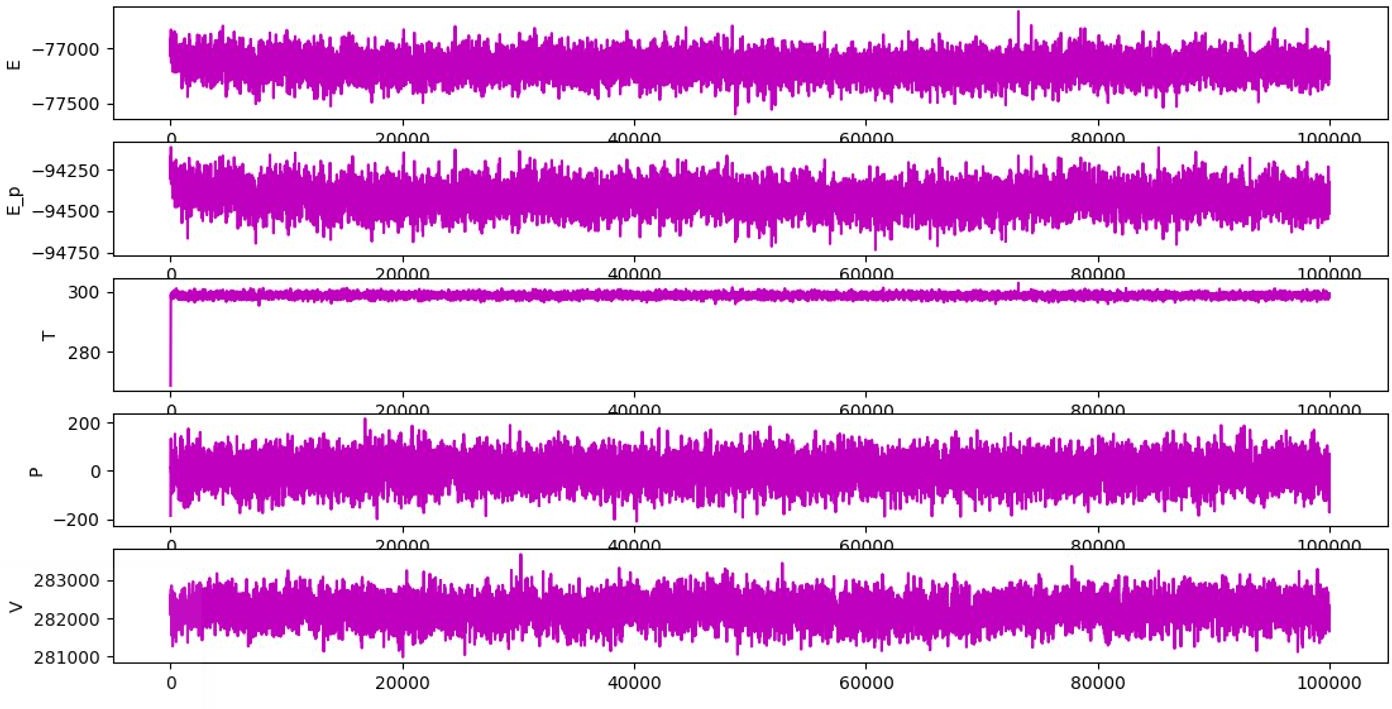


**Fig. S7**. (a) RMSD ligands of compounds **2** (red), **6** (green), **7** (orange), **9** (blue), and **10** (purple); (b) RMSF ligands of compound **2**

**
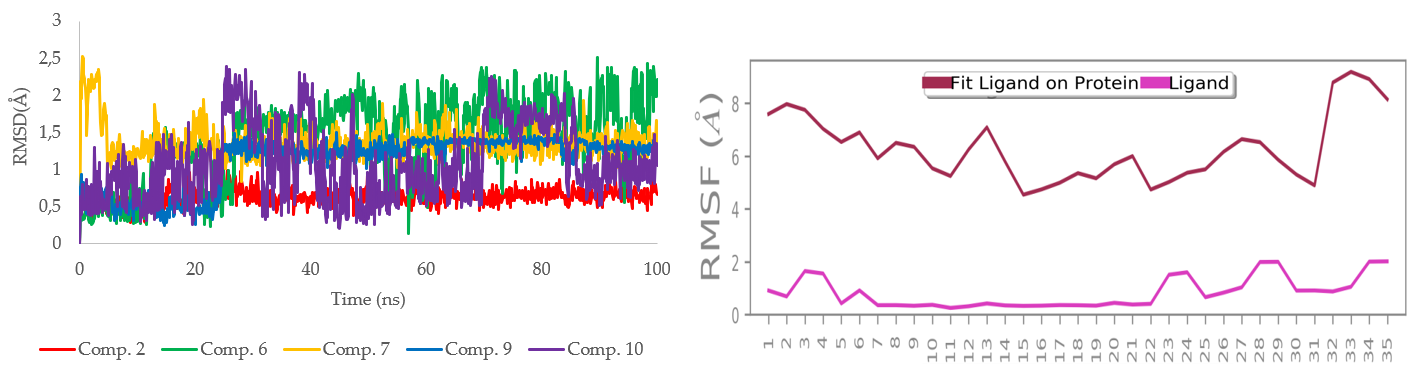
**

1. (b)

**Fig. S8. The** RMSF graph and residue contacts on the compound 2-NUDT5 complex in 100 ns MD simulation


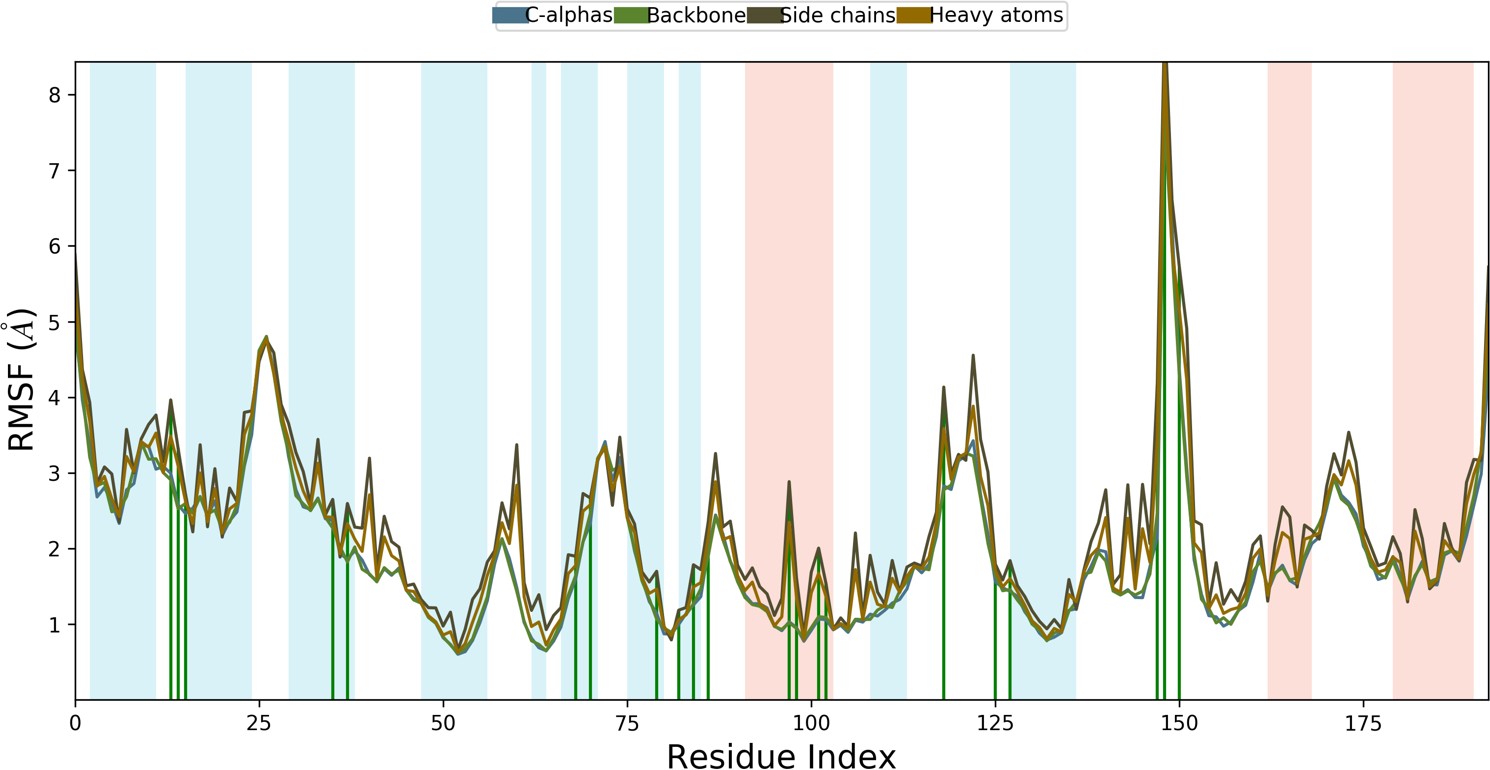


**Fig. S9. The** RMSF graph and residue contacts on the compound 7-NUDT5 complex in 100 ns MD simulation


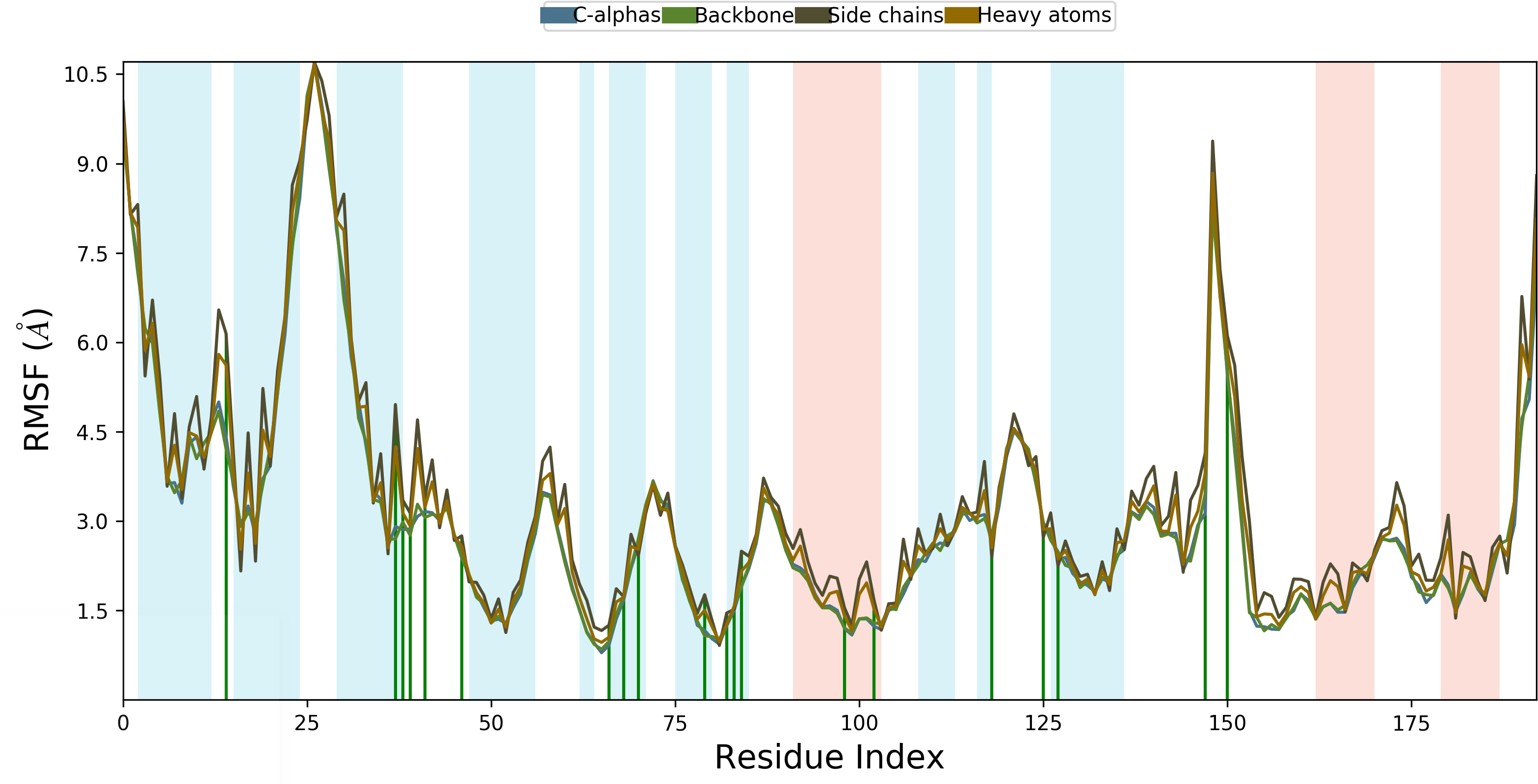


**Fig. S10. The** RMSF graph and residue contacts on the compound 9-NUDT5 complex in 100 ns MD simulation


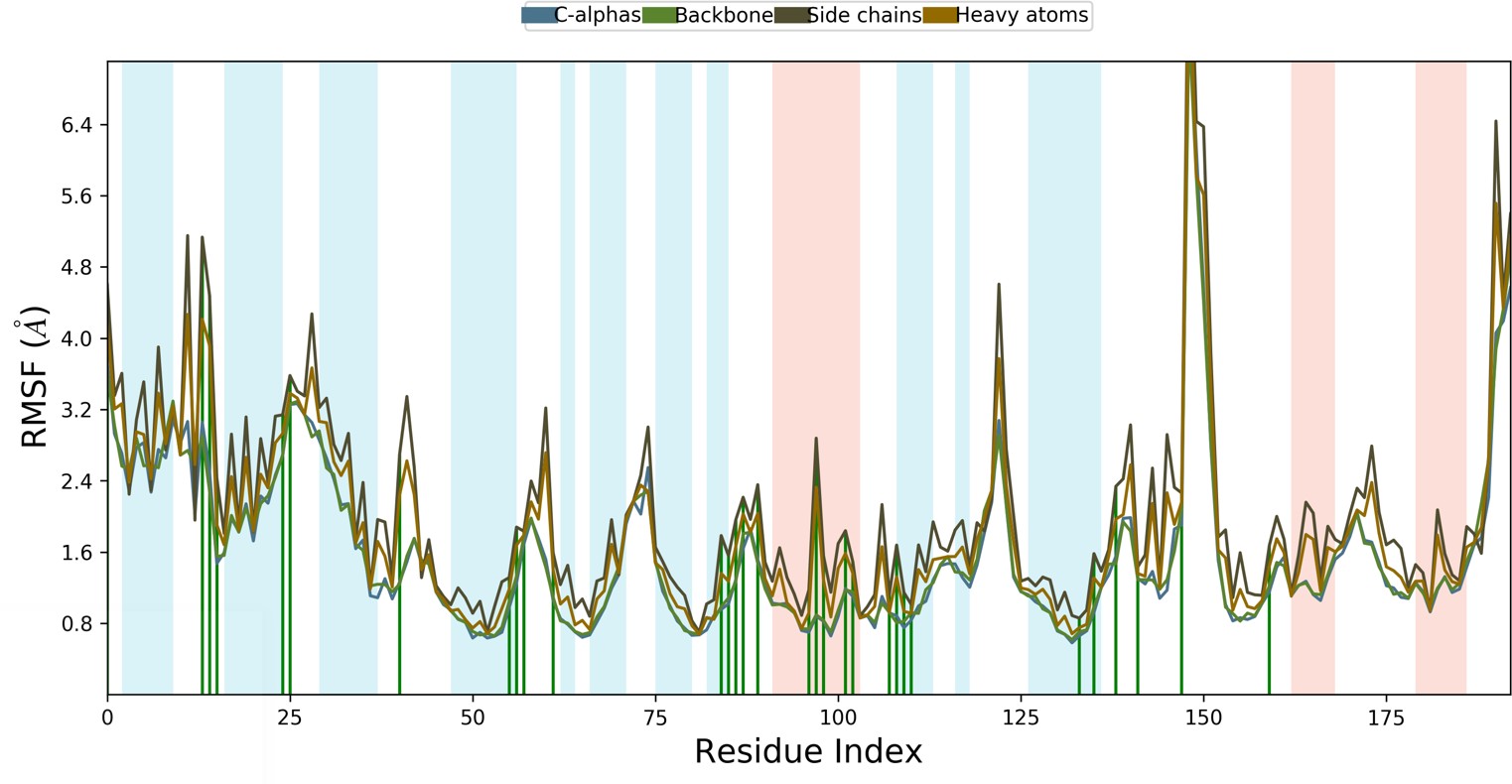


**Fig. S11. The** RMSF graph and residue contacts on the compound 10-NUDT5 complex in 100 ns MD simulation


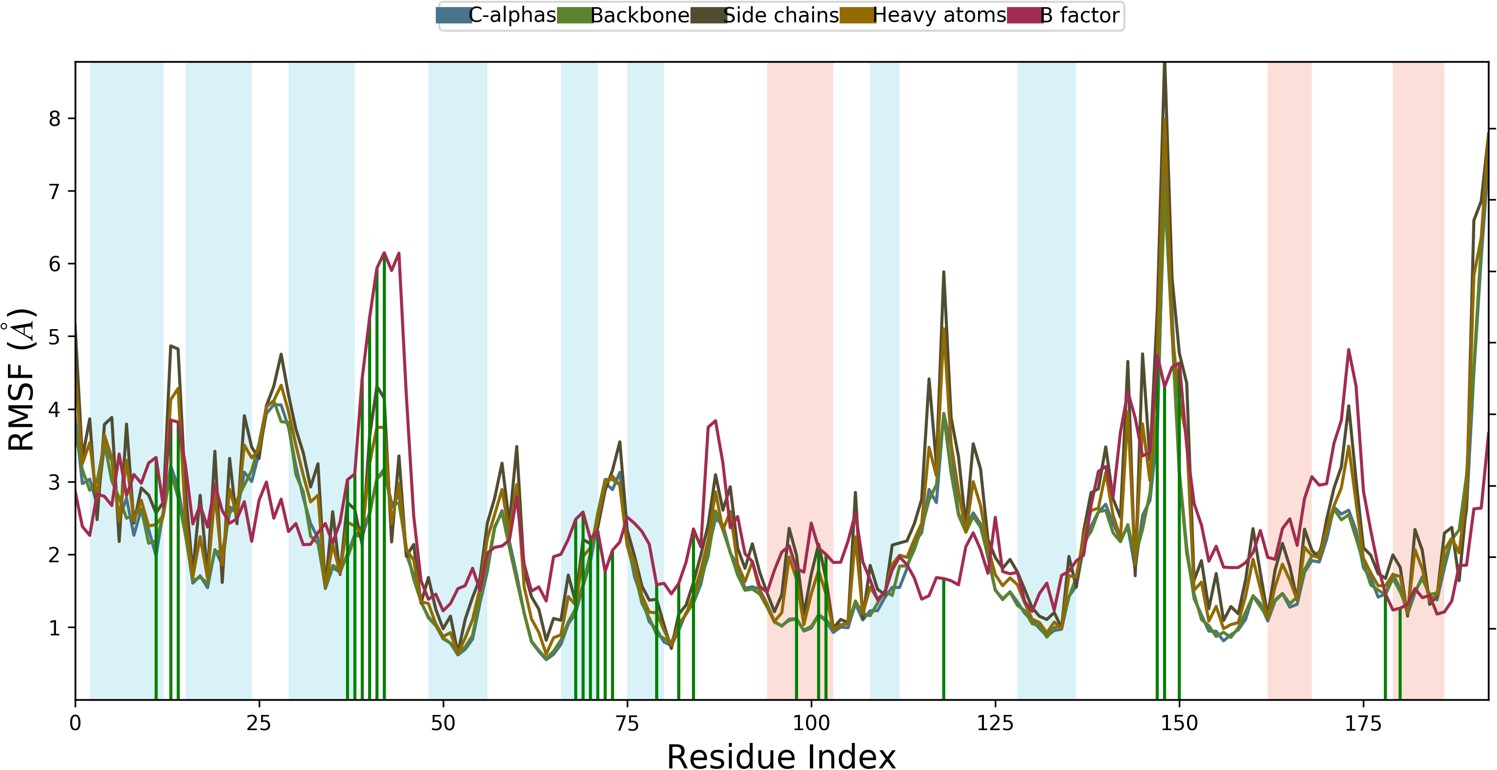


**Fig. S12.** The histogram bar chart showing the contact residues in compound 2-NUDT5


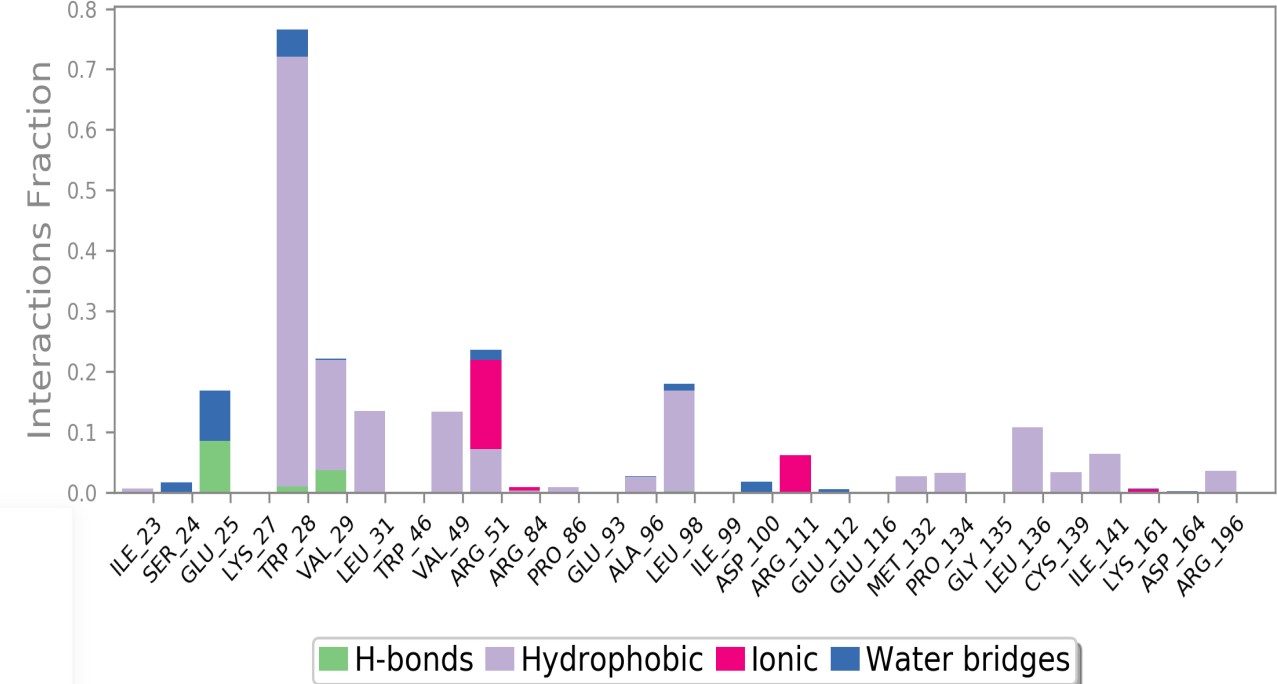


**Fig. S13.** The histogram bar chart showing the contact residues in compound 7-NUDT5


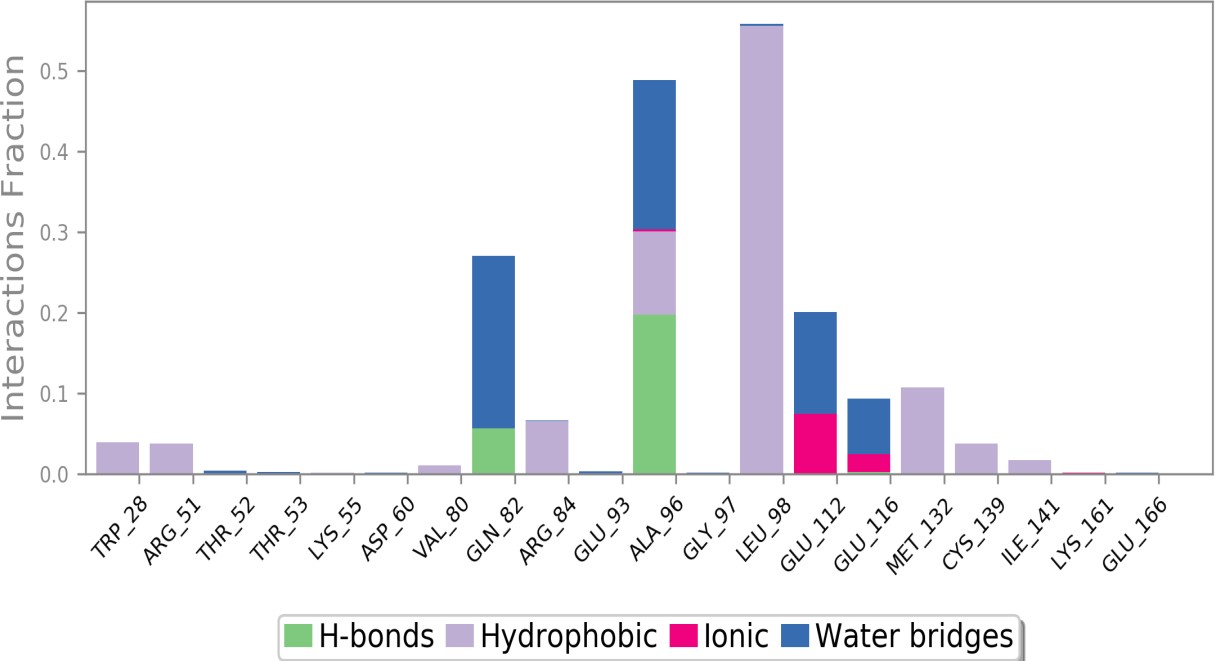


**Fig. S14.** The histogram bar chart showing the contact residues in compound 9-NUDT5


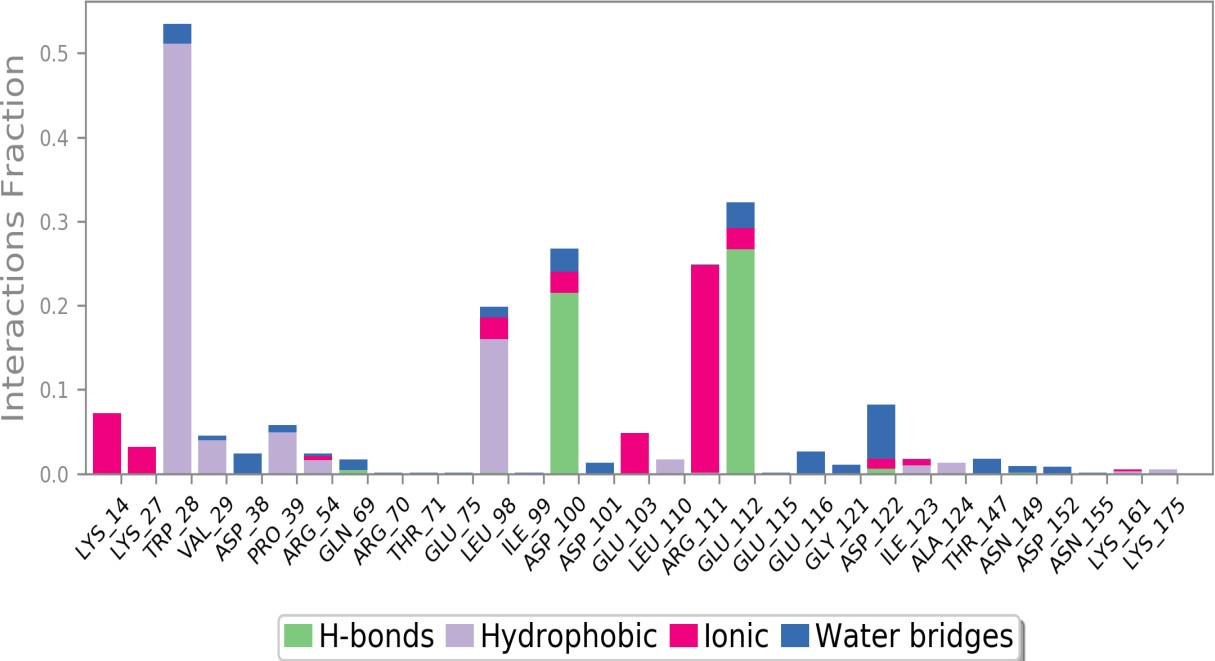


**Fig. S15.** The histogram bar chart showing the contact residues in compound 10-NUDT5


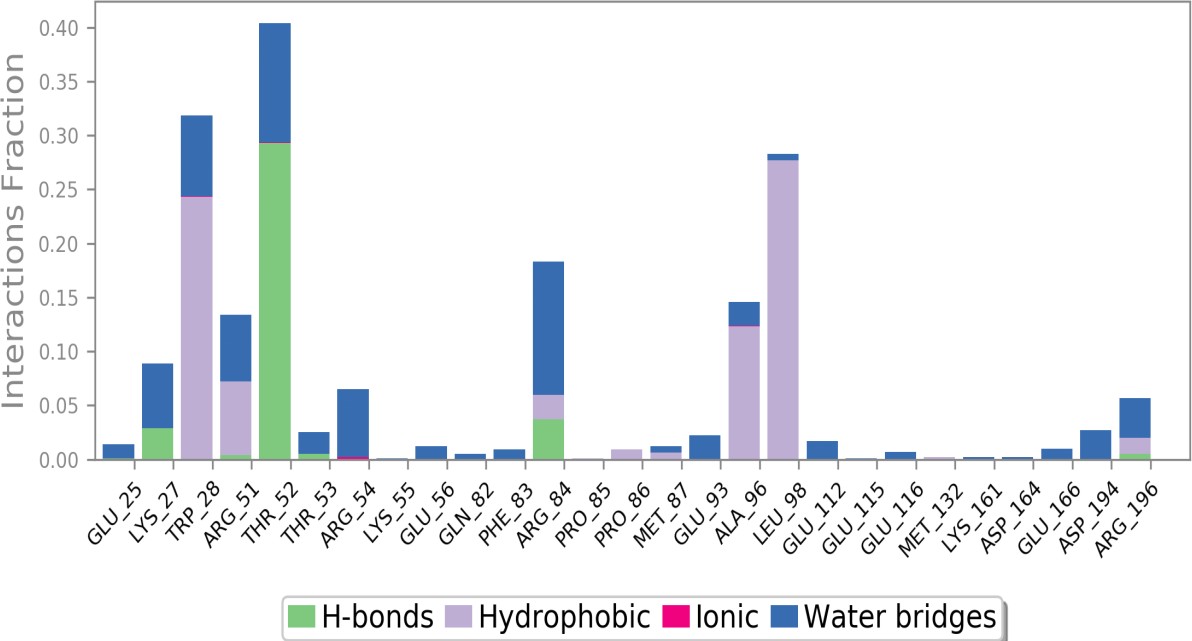


**Fig. S16.** Timeline representation of residue contacts and interaction of compound 2-NUDT5 in 100 ns

MD simulation


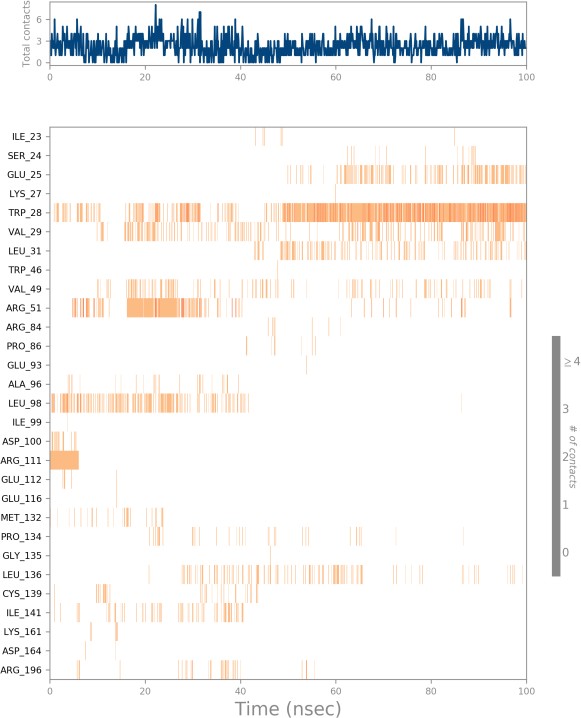


**Fig. S17.** Timeline representation of residue contacts and interaction of compound 7-NUDT5 in 100 ns MD simulation,


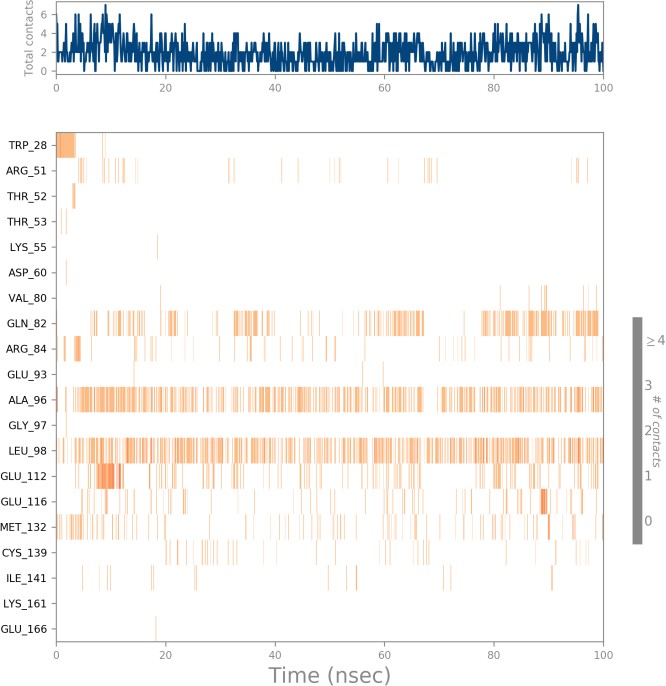


**Fig. S18.** Timeline representation of residue contacts and interaction of compound 9-NUDT5 in 100 ns MD simulation


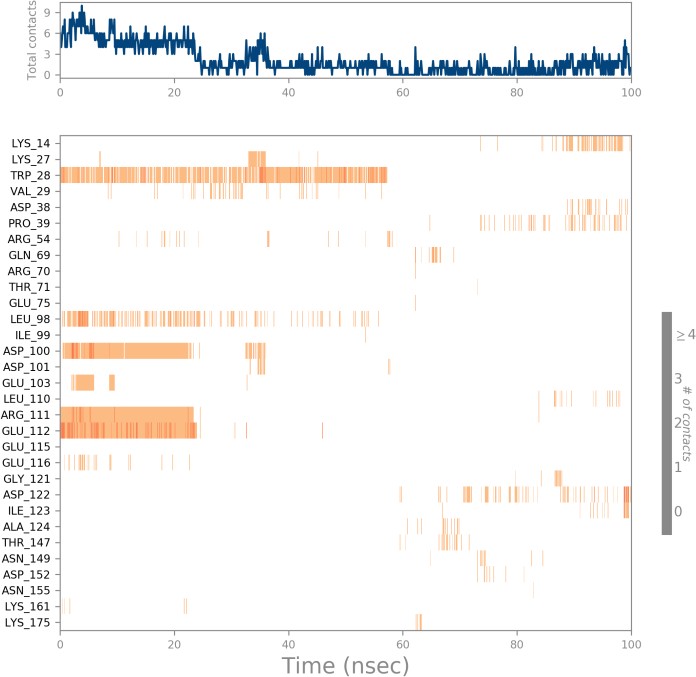


**Fig. S19.** Timeline representation of residue contacts and interaction of compound 10-NUDT5 in 100 ns MD simulation


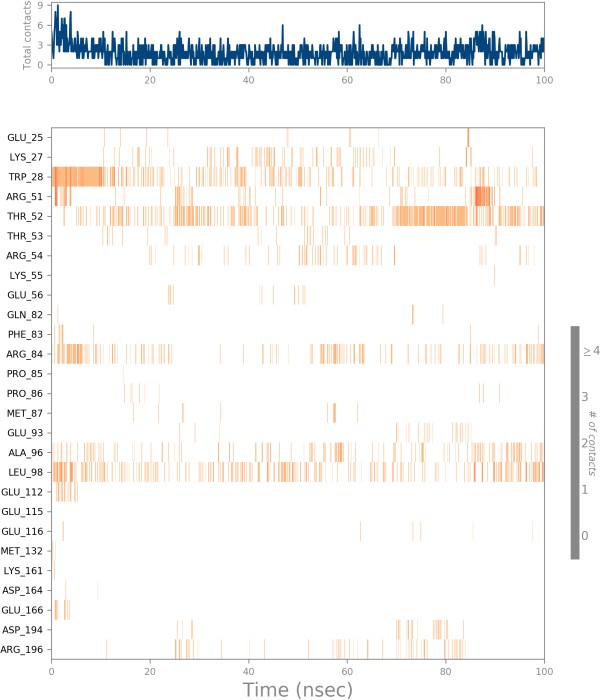


**Fig. S20**. Timeline representation of residue contacts and interaction of compound **6**-NUDT5 in 100 ns MD simulations,


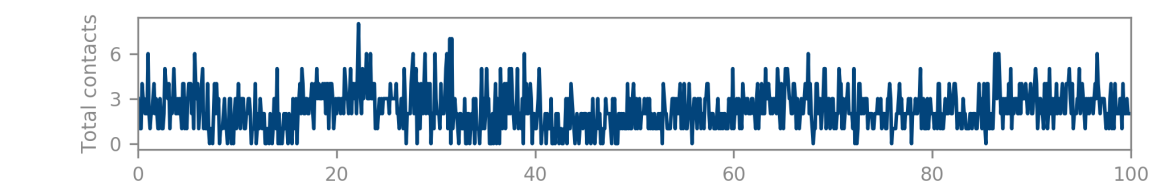

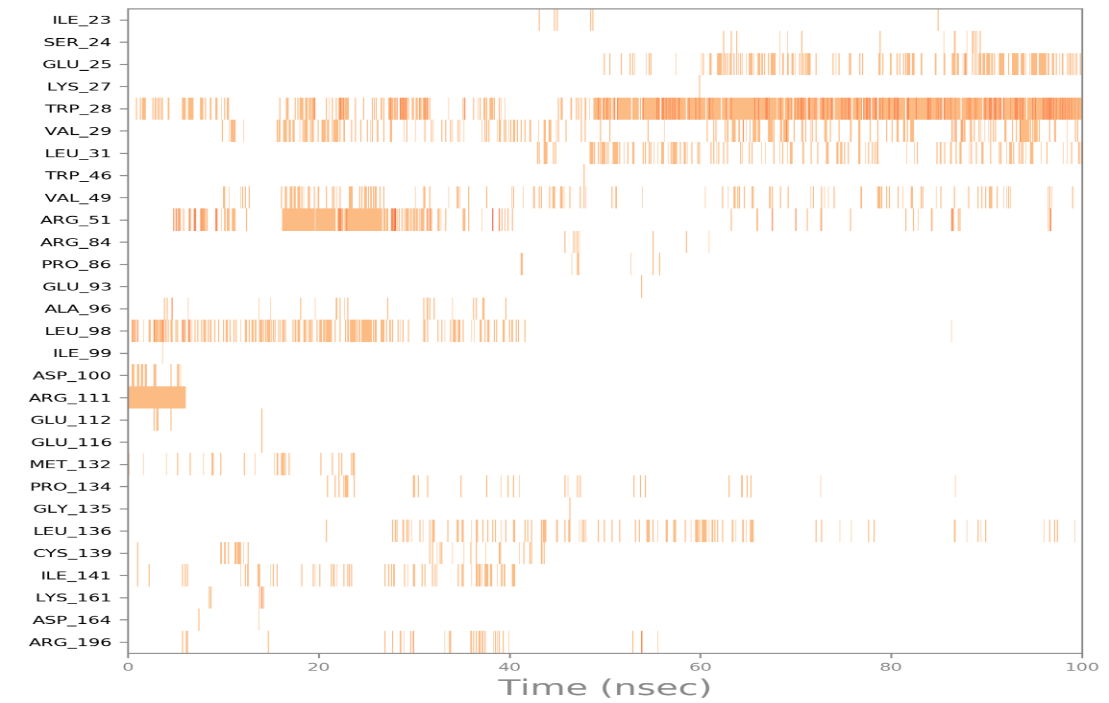

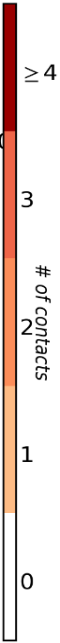


**Fig. S21**. Ligand properties during 100 ns simulations for compound **6**: (A) ligand RMSD (root mean square deviation), (B) radius of gyration (rGyr), (C) NS34, (D) molecular surface area (MolSA), (E) solvent accessible surface area (SASA), and (F) polar surface area (PSA),

**
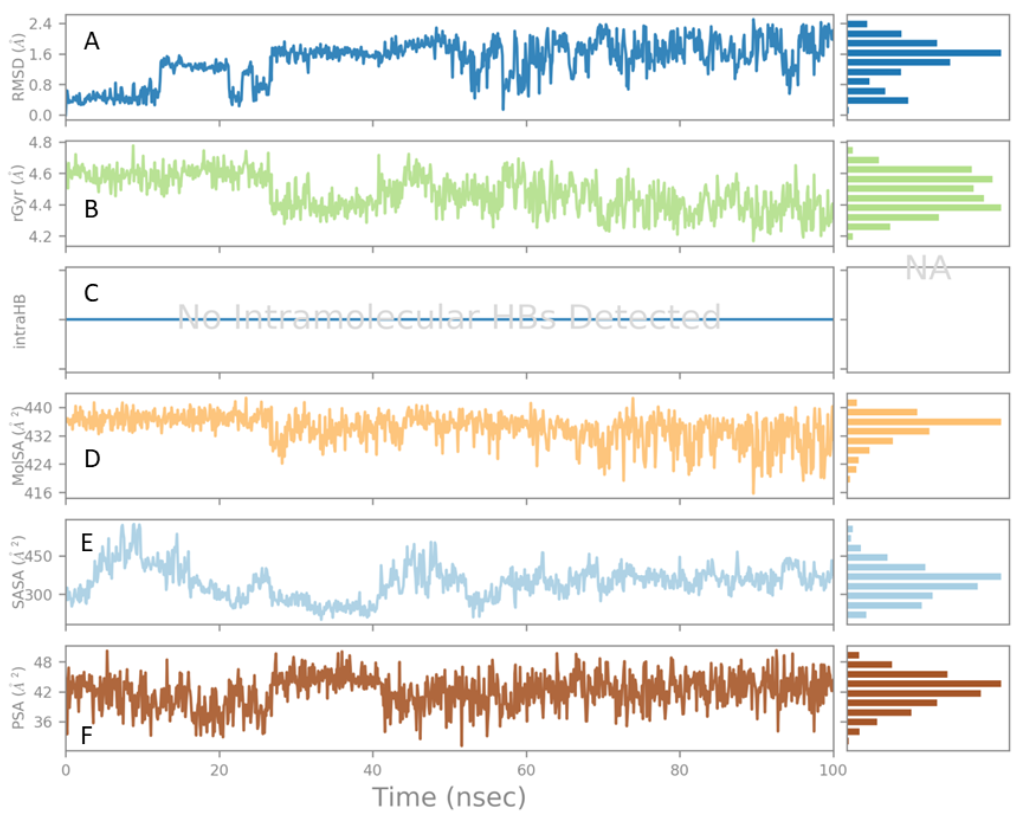
**

**Fig. S22**. Ligand properties during 100 ns simulations for compound 2: (A) ligand RMSD, root mean square deviation, (B) radius of gyration (rGyr), (C) NS34, (D) molecular surface area (MolSA), (E) solvent accessible surface area (SASA), and (F) polar surface area (PSA).


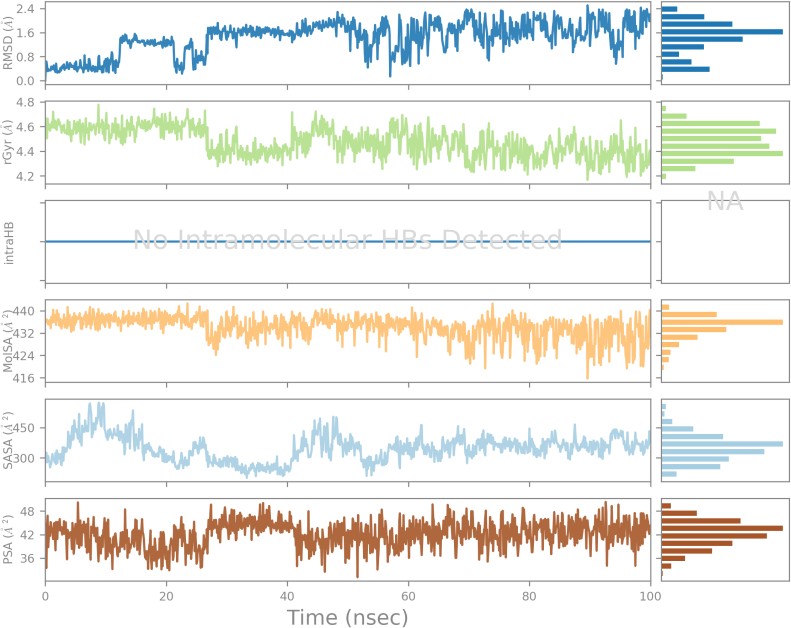
A B C D E F

**Fig. S23**. Ligand properties during 100 ns simulations for compound 7: (A) ligand RMSD, root mean square deviation, (B) radius of gyration (rGyr), (C) NS34, (D) molecular surface area (MolSA), (E) solvent accessible surface area (SASA), and (F) polar surface area (PSA).


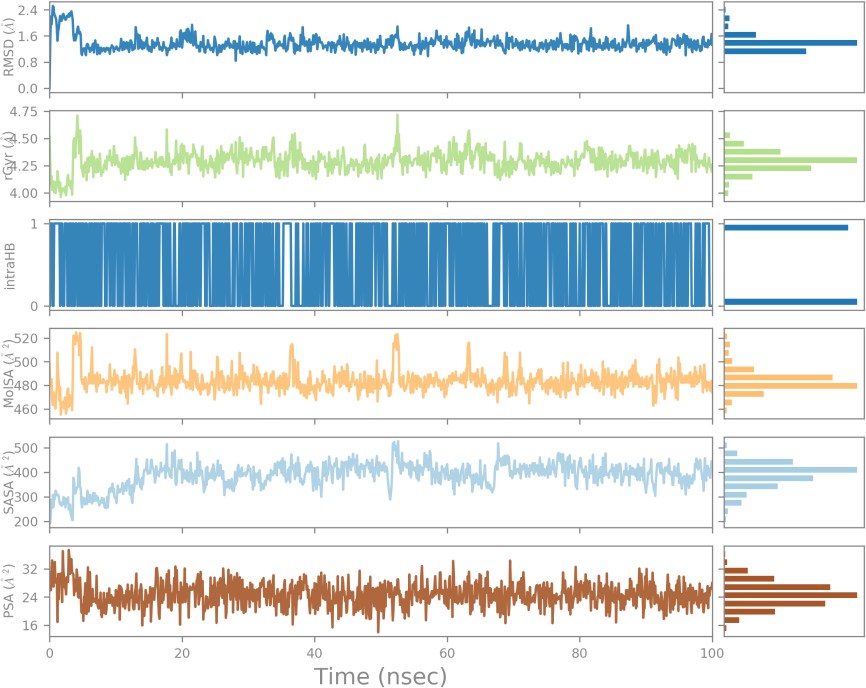
A B C D E F

**Fig. S24** Ligand properties during 100 ns simulations for compound 9: (A) ligand RMSD, root mean square deviation, (B) radius of gyration (rGyr), (C) NS34, (D) molecular surface area (MolSA), (E) solvent accessible surface area (SASA), and (F) polar surface area (PSA).


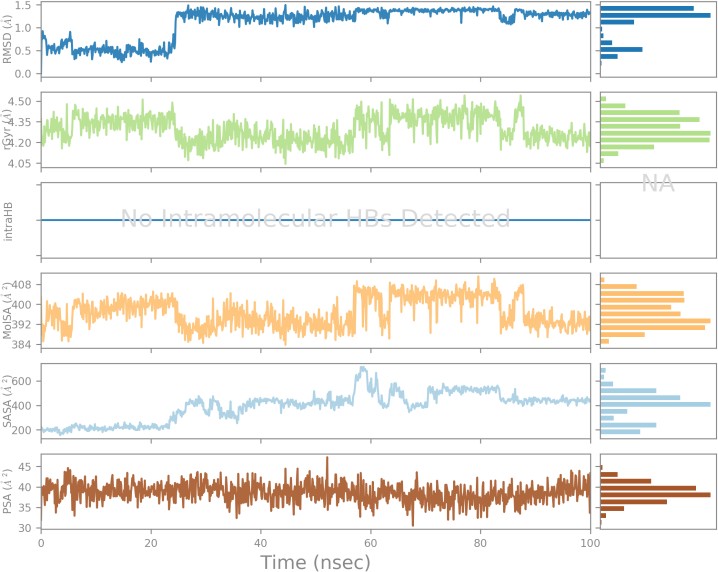


A B C D E F


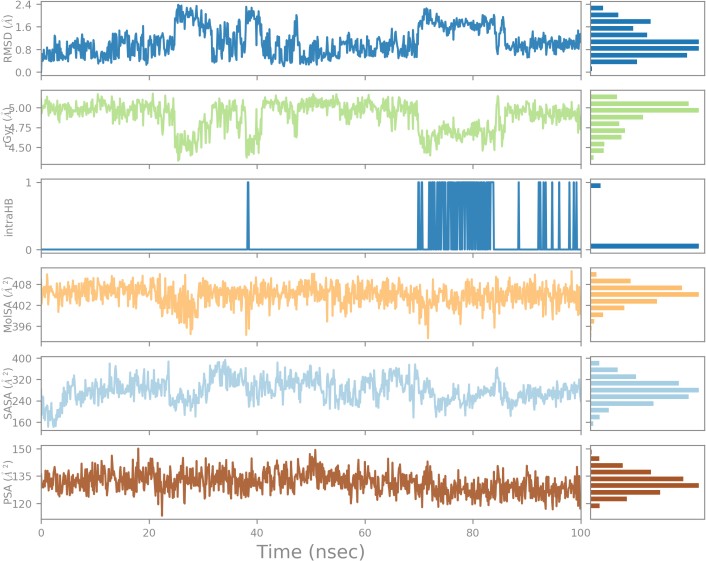
**Fig. S25**. Ligand properties during 100 ns simulations for compound 10: (A) ligand RMSD, root mean square deviation, (B) radius of gyration (rGyr), (C) NS34, (D) molecular surface area (MolSA), (E) solvent accessible surface area (SASA), and (F) polar surface area (PSA).

A B

C D E F
